# Neurovascular unit disruption and blood–brain barrier leakage in MCT8 deficiency

**DOI:** 10.1186/s12987-023-00481-w

**Published:** 2023-11-03

**Authors:** Marina Guillén-Yunta, Víctor Valcárcel-Hernández, Ángel García-Aldea, Guadalupe Soria, José Manuel García-Verdugo, Ana Montero-Pedrazuela, Ana Guadaño-Ferraz

**Affiliations:** 1 Laboratory of Thyroid Hormones and CNS, Department of Endocrine and Nervous System Pathophysiology, Instituto de Investigaciones Biomédicas ‘Alberto-Sols’, Consejo Superior de Investigaciones Científicas (CSIC), Universidad Autónoma de Madrid (UAM), C/ Arturo Duperier 4, 28029 Madrid, Spain; 2 Laboratory of Surgical and Experimental Neuroanatomy, Faculty of Medicine and Health Sciences, Institute of Neurosciences, University of Barcelona, Barcelona, Spain; 3 Laboratory of Comparative Neurobiology, Cavanilles Institute of Biodiversity and Evolutionary Biology and Department of Cellular Biology, University of Valencia and CIBERNED-ISCIII, Valencia, Spain

**Keywords:** Thyroid hormones, MCT8 deficiency, Thyroid hormone transporters, Blood–brain barrier, Neurovascular unit, Rare disease

## Abstract

**Background:**

The monocarboxylate transporter 8 (MCT8) plays a vital role in maintaining brain thyroid hormone homeostasis. This transmembrane transporter is expressed at the brain barriers, as the blood–brain barrier (BBB), and in neural cells, being the sole known thyroid hormone-specific transporter to date. Inactivating mutations in the MCT8 gene (*SLC16A2*) cause the Allan-Herndon-Dudley Syndrome (AHDS) or MCT8 deficiency, a rare X-linked disease characterized by delayed neurodevelopment and severe psychomotor disorders. The underlying pathophysiological mechanisms of AHDS remain unclear, and no effective treatments are available for the neurological symptoms of the disease.

**Methods:**

Neurovascular unit ultrastructure was studied by means of transmission electron microscopy. BBB permeability and integrity were evaluated by immunohistochemistry, non-permeable dye infiltration assays and histological staining techniques. Brain blood-vessel density was evaluated by immunofluorescence and magnetic resonance angiography. Finally, angiogenic-related factors expression was evaluated by qRT-PCR. The studies were carried out both in an MCT8 deficient subject and *Mct8/Dio2*KO mice, an AHDS murine model, and their respective controls.

**Results:**

Ultrastructural analysis of the BBB of *Mct8/Dio2*KO mice revealed significant alterations in neurovascular unit integrity and increased transcytotic flux. We also found functional alterations in the BBB permeability, as shown by an increased presence of peripheral IgG, Sodium Fluorescein and Evans Blue, along with increased brain microhemorrhages. We also observed alterations in the angiogenic process, with reduced blood vessel density in adult mice brain and altered expression of angiogenesis-related factors during brain development. Similarly, AHDS human brain samples showed increased BBB permeability to IgG and decreased blood vessel density.

**Conclusions:**

These findings identify for the first time neurovascular alterations in the MCT8-deficient brain, including a disruption of the integrity of the BBB and alterations in the neurovascular unit ultrastructure as a new pathophysiological mechanism for AHDS. These results open a new field for potential therapeutic targets for the neurological symptoms of these patients and unveils magnetic resonance angiography as a new non-invasive in vivo technique for evaluating the progression of the disease.

**Supplementary Information:**

The online version contains supplementary material available at 10.1186/s12987-023-00481-w.

## Background

Thyroid hormones (TH), T3 (3,5,3'-triiodo-L-thyronine) and T4 (3,5,3',5'-tetraiodo-L-thyronine or thyroxine), are essential for the proper development and function of the central nervous system (CNS). The thyroid gland mostly secretes T4, but T3 is the transcriptionally active form that modulates specific gene expression patterns, thus mediating most TH actions. T4 is metabolized to T3 by the action of the enzymes deiodinase type 1 (DIO1) and type 2 (DIO2). In the brain, 80% of T3 is generated by the action of the DIO2 enzyme present in astrocytes [1, 2]. From astrocytes, T3 is transported by poorly characterized mechanisms to the neurons and other neural cells of the brain parenchyma, where it binds to its nuclear receptors (TRα and TRβ) to regulate the expression of genes involved in myelination, neuronal and glial development, and function, among others. Therefore, a deficit of TH during the critical periods of the ontogeny of the CNS can cause irreversible neurological disorders [3].

To exert their actions, T3 and T4 have to cross the brain barriers and subsequently enter into their target neural cells via specific transmembrane transporters. Among these transporters, the monocarboxylate transporter 8 (MCT8) and the organic anion transporter polypeptide (OATP) 1C1 stand out for their physiological relevance in the CNS. MCT8 which is the sole known TH-specific transporter to date, is present both at the brain barriers and in neural cells [4–7]. Inactivating mutations in the *SLC16A2* gene, which encodes for MCT8, result in a rare X-linked disorder called Allan-Herndon-Dudley Syndrome (AHDS) or MCT8 deficiency. This complex disease is characterized by peripheral hyperthyroidism with elevated T3, associated with low T4 and 3,5’,3’-triiodothyronine or reverse T3, and normal or slightly elevated thyrotropin or TSH, and brain hypothyroidism, with low T3 and T4 in the brain [8]. The main problem for TH action in AHDS patients’ brains seem to derive from the restriction in TH entry into the brain parenchyma, and to a lesser extent, to the restricted entry of TH into neural cells, as other TH transporters could compensate for the absence of MCT8. This leads to neurological symptoms such as delayed neurological development, severe intellectual disability (IQ < 30), or central hypotonia with spastic paraplegia [9–12], along with severe histopathological alterations [8, 13]. TH transport across the BBB has a pivotal role for AHDS pathophysiology, as demonstrated by the expression of MCT8 in the human brain barriers along neurodevelopment [5, 6] and in different functional studies showing altered TH transport due to MCT8 inactivation using both in vivo [14] and in vitro models [15]. However, the pathophysiology of the syndrome concerning the integrity and permeability of the BBB, the main brain barrier involved in the direct availability of TH for neural cells [16, 17], has not been studied to date in human brain tissue.

The BBB is a highly selective barrier maintaining a unique microenvironment within the brain parenchyma. It regulates the traffic of molecules between the CNS and the peripheral tissues, preventing the entry of exogenous or hazardous molecules from the bloodstream into the CNS. The structural elements of the BBB include endothelial cells, tightly sealed between each other by tight junctions (TJ), adherens junctions, and a dense extracellular matrix surrounding the vessels; the pericytes, contractile cells that help maintain the vascular tone of the vessels; and the astrocyte end-feet (AEF), with specific transporters contributing to the precise regulation of molecule trafficking across the BBB [18]. These cellular components, including endothelial cells, pericytes, astrocytes as well as neighboring neurons and microglia form a dynamic functional unit known as the neurovascular unit (NVU) [19].

T3 has been shown to directly regulate the expression of certain adherens junction proteins such as E-Cadherin or α- and β-catenins, by modulating their transcription [20]. In addition, it is noteworthy that T4, through extragenomic actions, specifically contributes to the regulation of the expression of angiogenic transcription factors such as VEGF, bFGF, or Ang-2, as well as the proangiogenic cytokines CXCL2 and CXCL3. This regulatory effect occurs by T4 binding to the αvβ3 integrin at the plasma membrane [21, 22]. In addition, it has also been suggested a possible role of T3 in the modulation of some of these genes by means of genomic actions [23]. Therefore, dysregulation in the brain content of TH can lead to a dysfunction in the angiogenic process, and alterations in any component of the NVU might compromise the integrity of the BBB, with subsequent pathological consequences within the brain parenchyma.

While current research on the action of TH on the vascularization of the BBB remains limited, it has been shown that TH induce angiogenesis sprouting and capillary growth in the cardiac tissue of hypothyroid mice [24]. Additionally, a deficit of TH during early postnatal stages has been found to decrease vascular density and branching complexity in the CNS [23]. Moreover, recent work in this field corroborates that TH defects are correlated with perturbations on cerebral small vessel morphology [25]. Thus, we hypothesized the lack of function of MCT8 in the brain barriers, especially in the plasma membrane of the endothelial cells, has an impact on BBB integrity and functionality.

In this work, we have explored the structure and function of the BBB in human brain samples from an MCT8-deficient patient and in *Mct8/Dio2*KO mice, a valuable model for AHDS. Given that *Mct8*KO mice replicate the peripheral hyperthyroidism but do not show the neurological alterations present in AHDS patients due to the mouse-specific compensatory mechanism based on the alternative T4 transporter organic anion transporter 1c1 (OATP1C1) and the enzyme DIO2, the *Mct8/Dio2*KO mice represent a more faithful model for the disease. These mice replicate both the peripheral hyperthyroidism and the cerebral hypothyroidism characteristic of MCT8-deficient patients, with a significant reduction in the cerebral T3 content. Moreover, these mice also display locomotor abnormalities and alteration in different neural and myelin biomarkers [13, 26].

Our results showed a BBB disruption with loss of integrity and increased permeability in the *Mct8/Dio2*KO mice at postnatal day 90 (P90) and P180. Additionally, we have observed an increased BBB permeability in the human pathological brain samples, acknowledging a new paradigm in the study of this rare disease. These findings can be translated into potential new therapeutic targets and biomarkers associated with the neurological symptoms of the disease and may contribute to the development of therapeutic strategies aimed at improving the quality of life for MCT8-deficient patients.

## Materials and methods

### Human brain samples

Human brain tissues were obtained from necropsies from an 11-year-old MCT8-deficient male subject and a closely age-matched control subject without known brain pathology. The BRISQ guidelines for each human specimen [27] containing relevant information about their clinical traits and preservation methods can be found in Additional file 1: Table S1.

Tissue blocks from both subjects were fixed in formalin and embedded in paraffin using standard procedures. 7 μm thin sections were obtained in a microtome (Microm, HM 310) and adhered to glass slides pre-treated with Poly-L-Lysine solution (Sigma-Aldrich, P8920).

### Animals

All mice were housed at the Biomedical Research Institute “Alberto-Sols” under light- and temperature-controlled conditions of a 12:12 light–dark cycle at 22 ± 2 °C with ad libitum access to food and drinking water. *Mct8*KO mice were originally donated by Dr. Samuel Refetoff, from the University of Chicago. *Dio2*KO mice were originally donated by Dr. Valerie Galton, of the Dartmouth Medical School. The *Mct8/Dio2*KO mice were generated in the same C57BL/6 J genetic background by mating *Mct8*^+/y^/*Dio2*^−/−^ males and *Mct8*^−/+^/*Dio2*^−/−^ females as previously described [26]. As AHDS is an X-linked disease, only male mice were used for this study. Mice were genotyped with suitable primers for MCT8 and DIO2 genes following a tail DNA PCR using a protocol already established [14].

Tissue for histological procedures was obtained after anesthetizing the animals with ketamine (Imalgene 100 mg/mL, 21029) and medetomidine hydrochloride (DOMTOR, Esteve Veterinaria, 135089-1) and perfusion with 4% paraformaldehyde (Merk Millipore, 1.04005) in 0.1 M phosphate buffer saline (PBS). The brain was removed and post-fixed in the same solution for 24 h and was subsequently cryoprotected in 30% sucrose (SIGMA, 16104). Serial tissue sections of 25 μm were obtained using a cryostat (Leica CM1950) and stored at -80°C. For western blotting, the animals were perfused with PBS and the dissected tissues were stored at -80°C until used. For TEM procedures, mice were transcardially perfused with 2% paraformaldehyde and 2% glutaraldehyde (Electron Microscopy Sciences, 16210) in 0.1 M phosphate buffer (PB). Brains were collected and postfixed overnight in 2% paraformaldehyde and 2% glutaraldehyde in 0.1 M PB at 4 °C, then preserved in 0.05% sodium azide in 0.1 M PB at 4 °C and finally cut into 100 µm sagittal sections on a vibratome (Leica VT1000S)***.***

The experimental design for each procedure involving animal experimentation was carried out following the ARRIVE guidelines [28]. For details see Additional file 1.

### Transmission electron microscopy (TEM)

Mice brain slices were postfixed in 2% OsO_4_ in 0.1 M PB (90 min at RT) and stained in 2% uranyl acetate stained (150 min at 4 °C in the dark), ethanol dehydrated, immersed in propylene oxide (Lab Baker, Deventry, Holland) and embedded overnight in Araldite (Durcupan, Fluka). Semithin sections (1.5 µm) stained with toluidine blue were used to select a representative section of all layers of the cerebral cortex. Ultrathin sections (60 nm) were cut using an ultramicrotome (Leica Ultracut S), put onto 200 mesh copper grids, and counterstained with lead citrate.

Images were then obtained with a transmission electron microscope Jeol Jem1010 (Jeol, Tokyo, Japan) equipped with a Gatan SC200 digital camera (Gatan Inc., Pleasanton, CA).

We analyzed all the blood vessels within an approximate area of 300 × 60 µm in the motor cortex, including all layers from pia matter to layer VI in a sagittal section of 100 µm at lateral 1.08 mm. A total of 80 to 100 blood vessels were photographed per group (15 to 25 per animal). Images at 40,000X and 80,000X magnification were taken to study in detail the integrity of the TJ, cytoplasm cohesion, basal lamina protrusion status, astrocytes, and for the presence or absence of transcytotic vesicles in the blood vessels. Images were analyzed using Image J^®^ software. The percentage of abnormal components of the BBB was calculated as follows:

$$\%\;of\;abnormal\;structures=\frac{\#\;of\;vessels\;with\;aberrant\;components}{total\;number\;of\;vessels}$$

The average basal lamina width was measured as the mean of 12 randomized measures of the basal lamina width of each blood vessel taken at 40,000 × magnification.

### Western Blot (WB)

Frozen cortical brain tissue was homogenized in RIPA buffer (50 mM Hepes, 1% Triton-X, 150 mM NaCl, 10% glycerol, 1% deoxycholate, 0.1% SDS) with protease inhibitors (Complete Mini protease inhibitor cocktail tablets, 11,836,153,001, Roche) with a polytron (Polytron System PT 1200 E, Kinematica), incubated at 4 °C for 30 min in a spinning wheel, then centrifuged at 4 °C and 14,000 rpm for 15 min and the supernatant was collected for protein measurement using the Bradford method (Bio-Rad Protein Assay, 500–0006). Protein samples were run in a 7–12% acrylamide/bis-acrylamide gel (MB04501, Nzytech) and then transferred into a nitrocellulose membrane (10600002, Amersham Protran 0.45 µm NC, GE Healthcare). The membrane was blocked in tween tris buffered saline-BSA 4% (SIGMA, A9647) for 1 h at RT, and incubated with the primary antibodies (zonula occludens 1 (ZO-1), 1:1000, sc-8146; α-Tubulin, 1:3000, sc-32293; occludin, 1:1000, sc-133256; Gapdh, 1:1000; sc-32233) at 4 °C overnight on a seesaw. After several washes in Tween-Tris buffered saline, the membrane was incubated with the appropriate secondary antibody (donkey anti-goat IgG-HRP, 1:5000, sc-2020, goat anti-mouse IgG-HRP. 1:5000, sc-2005) for 1 h at RT. The immune signal was revealed by an enhanced chemiluminescence kit (RPN2209, GE Healthcare), using a Fusion Solo S Chemiluminescence Reader (Vilber) and the EvolutionCapt software. The densitometry of the band intensity was determined with Image J® software.

### Immunohistochemistry (IHC)

For paraffin-embedded human brain tissues, a deparaffination step was done with Xilol (VWR Chemicals, 28973.294), 100°, 95°, 70° and 50° ethanol solutions and distilled water, followed by an antigen retrieval step (Dako, GV804) following the manufacturer’s instructions. Endogenous peroxidase activity was blocked for 15 min in the dark with 3% H_2_O_2_ (EMSURE, K48386109648), and 10% methanol (EMSURE, 1060092500) in PBS. Non-specific binding of the primary antibody was performed with a blocking solution (0.1% Triton-X100 (Merck Millipore, 1.08603), 4% BSA (SIGMA, A9647) 5% normal horse serum (Vector laboratories, S-2000-20) in PBS) for 1 h at room temperature (RT). Tissue sections were incubated with the primary antibody (hIgG 1:300, ABCAM, ab109489 for human studies) in 0.1% Triton-X100, 4% BSA, and 1% serum in PBS overnight at 4 °C. Tissues were then incubated with a biotinylated secondary antibody (goat anti-rabbit IgG biotinylated 1:200, BA-1000, Vector Laboratories for human studies and horse anti-mouse IgG biotinylated, 1:200, BA-2001, Vector Laboratories for mice studies) in 0.1% Triton-X100, 4% BSA, 1% serum for 1 h at RT. The immune signal was amplified using the ABC kit for 1 h at RT in the dark (Thermo Scientific, 32050) and immunostaining was developed with diaminobenzidine (DAB, Sigma Aldrich, D5637) and 0.01% H_2_O_2_. Samples were then dehydrated with various steps in 50°, 70°, 95°, and 100° ethanol solutions and Xylol and finally covered and preserved with hydrophobic mounting medium Depex (SIGMA, 06522).

For free-floating mice tissue sections, the previous deparaffination step was not necessary, and the primary antibody step was omitted as the secondary antibody used was able to detect the mouse IgG.

For IHC experiments, the procedures were performed in parallel for the control and experimental tissue to avoid methodological differences. Also, negative control without a primary antibody was carried out to account for unspecific signals.

The tissue was visualized with a bright-field Nikon Eclipse 80i microscope, and the photographs were obtained with a Nikon DSFi1 digital camera and the Niss Elements Imaging Software (version 4.20.01) using 10 × (NA 0.30), 20 × (NA 0.50), 40 × (NA 0.75) or 100 × (NA 1.30) objectives.

For IgG immunosignal quantification of the human samples, the specific IgG signal surrounding blood vessels in the human motor and sensory cortex in both the parenchyma and the subcortical white matter was quantified with ImageJ following a well-stablished protocol [13]. In brief, after eliminating the IgG signal from the lumen of the blood vessel, we measured the percentage of area occupied by immunopositive signal as well as the mean immunopositive signal mean value (mean grey). Then, we calculated the AI index (% area immunolabeled multiplied by the mean gray value), and results were expressed as fold changes normalized by the value of the control sample. For the quantification of IgG in mice, three 20 × magnification images were captured from three brain tissue sections from Bregma 1.42 mm to Bregma -0.46 mm for each animal (n = 4) covering the motor and somatosensory cortex.

### BBB permeability assessment by SF and EB infiltration assays

*Evans blue (EB):* a 20 mg/mL EB solution (SIGMA, E2129) diluted in saline was administered to the mice at a dose of 80 mg/kg body weight by tail-vein injection. After 90 min, mice were transcardially perfused with 15 mL of saline and brains were collected, weighed, photographed, and then incubated in 1 mL of formamide solution (SIGMA, 47671) per 200 mg of tissue for 24 h at 56 °C and 150 rpm shaking. Formamide extracts were collected and the EB concentration was measured at 620 nm in a VERSAmax™ Tunable Microplate Reader (Molecular Devices, 89429-538) using the SOFTmax® PRO software (Version 3.0). EB concentration in each sample was calculated by interpolation of the mean values of three replicates from the standard curve.

*Sodium fluorescein (SF):* a 5% SF solution (SIGMA, F6377) diluted in saline was administered to the mice at a concentration of 200 µg/g body weight by tail-vein injection. After 4 h, mice were transcardially perfused with 15 mL of saline and brains were collected, weighed, photographed, and frozen at − 80 °C uniformly over an iron plate. Tissues were lyophilized at 0.02 mBar and − 80 °C in a Freeze Dryer (Telstar Cryodos). Tissue water content was calculated as the difference in weight between fresh and lyophilized tissue. Lyophilized samples were then incubated in 10 µL of formamide solution (SIGMA, 47671) per mg of tissue for 24 h at 56 °C and 150 rpm shaking. Formamide extracts were collected and diluted 1:3 in absolute ethanol to increase the fluorescent sensitivity and the SF fluorescence was measured in a FLUOstar OPTIMA fluorimeter Microplate Reader (BMG Labtech) with 440 nm excitation/525 nm emission filters, and data were obtained using the OPTIMA MARS Data Analysis software. SF concentration in each sample was calculated by interpolation of the mean of three replicates from the standard curve. The data for each experiment were normalized to a value of 1.0 AU for the mean of the WT group.

### Histological staining procedures

*Hematoxylin & Eosin (H&E).* Mice brain frozen tissue sections were stained with Carazzi Hematoxylin (Fluka, 51260) for 4 min, cleared in tap water and distilled water for 5 min and the signal was differentiated with 1-min steps in 70° and 95° ethanol solutions. Then, samples were stained with Eosin Y solution with phloxine (SIGMA, HT110332) for the detection of acute or fresh microhemorrhages for 30 s and the signal was differentiated for 20 s in a 70° ethanol solution. Finally, clearance with 1-min 100° ethanol and 6 min Xylol solutions was carried out and the samples were covered and preserved with hydrophobic mounting medium Depex.

*Prussian Blue.* After blocking endogenous peroxidases of the mice frozen brain tissue sections as specified in the IHC section, the tissues were incubated for 30 min with a 1:1 mix of 2% potassium hexacyanoferrate (II) trihydrate (SIGMA, P9387) and 3.7% HCl (Merck Millipore, 1.00317), followed by distilled water washing. The signal was revealed by a 15-min incubation in DAB 0.005 mg/mL solution followed by 10-min incubation in DAB 0.005 mg/mL with 3% H_2_O_2_. Then, tissue sections were counterstained with Carazzi Hematoxylin, dehydrated, and coverslipped as described in the IHC section. Prussian Blue histological staining accounts for sub-acute microbleeds as the ferrocyanide of the Prussian Blue reacts with the hemosiderin of the degraded hemoglobin of the lysed red blood cells.

The microscope, objectives, and camera software used for analyzing all histological staining procedures were as described in the IHC section. The quantification of the number of acute and subacute microbleeds was carried out in the whole brain parenchyma of serial brain tissue sections from Bregma 2.62 mm to Bregma − 3.88 mm, interspaced 25 µm between each other to sample a representation of the whole brain parenchyma.

### Blood vessel labeling

For paraffin-embedded human brain tissues, a deparaffination step was performed as described in the IHC section. Sections were blocked using a blocking solution (4% BSA, 3% normal horse serum in PBS) for 1 h at room temperature (RT). Tissue sections were incubated with blood vessel-specific glycoprotein (Daylight 594 *Ulex Europaeus* Agglutinin I (UEA-I), 1:50, Vector Laboratories, DL-1067-1 for human samples or Daylight 594 Tomato-Lectin, 15 µg/µL, Vector Laboratories, DL-1177-1 for mice samples) in 4% BSA, 1% serum in PBS overnight at 4 °C. Tissues were then incubated with DAPI (1:1000, Invitrogen, D1306) for 15 min at RT for nuclei staining and then covered and preserved with FluorSave hydrophilic medium (Merck, 345789).

The tissue was visualized and blood vessel images were taken with a Zeiss LSM710 Laser Scanning Microscope (Carl Zeiss).

The specific Daylight 594 UEA-I signal of human brain tissue samples and Tomato-Lectin signal of mice brain tissue samples were quantified with ImageJ software. In brief, for human tissues, maximal intensity projection images from a tile stack of five different planes covering the whole cortex from the meninges to the beginning of the white matter obtained with a 25 × magnification objective. For mice tissues, maximal intensity projection images from a stack of three different planes obtained from Bregma 1.42 mm to Bregma − 0.46 mm for each animal (n = 4) with a 25 × magnification objective were used. To eliminate unspecific signal, a threshold procedure was applied and then we measured the percentage of area occupied by red fluorescent signal corresponding to blood vessels stained with UEA-I or Tomato-Lectin relative to total image area. Results were expressed as fold changes normalized by the mean of the control sample/group.

### Magnetic resonance angiography (MRA)

Animals were anesthetized with isoflurane and a 2D Time of Flight (TOF) MRA without contrast agent was acquired in a Biospec spectrometer 7 T (Bruker Biospec 70/16 US (AV3); model number 1P BAP 70/16; serial number: S10051840, Bruker Biospin MRI GmbH, Ettlingen, Germany) with the following parameters: TE: 2.6 ms, TR: 15 ms, FA: 80°, 80 slices of 0.3 mm without gap, matrix 256 × 256 and FOV 23 × 23 mm.

Whole-head MRA images were obtained and denoised to diminish the possible artifacts before image processing. Then, a segmentation step was carried out to delimit the brain area and avoid cranial blood vessels with the Insight ToolKit (ITK-SNAP) free-licensed segmentation software [29]. Finally, a maximal intensity projection (MIP) was obtained with a custom-made Image J® plugin. The cerebral blood vessel-covered area was obtained as follows: (blood vessel-specific area / segmented whole brain area) [30]. Segmented whole brain area was obtained manually with the Image J® region of interest (ROI) and ROI Manager tools, and the blood vessel-specific area was obtained with a first step of the Image J® Threshold tool to eliminate the unspecific parenchymal signal followed by the use of the ROI and ROI Manager tools. A Pseudocolor Image Look-Up Table (LUT) Image J® map plugin was run to elaborate the heatmap images.

### Quantitative real-time PCR (qRT-PCR)

RNA was isolated from single hemi-cortices of embryonic day 15.5 (E15.5) and E18.5 WT and *Mct8/Dio2*KO embryos and treated with DNAse using columns. The integrity of the obtained mRNA was evaluated by RNA Nano Chip RNA Integrity Number (RIN) in a 2100 Bioanalyzer (Agilent, 5067–1511). 250 ng of mRNA were retrotranscribed to cDNA using a High-Capacity cDNA Reverse Transcription Kit (Applied Biosystems, 4368813). Then, a sample of 50 ng/well of the cDNA was used in triplicates for the qRT-PCR using the Power SYBR Green PCR Master Mix (Applied Biosystems, 4367659) on a l QuanStudio 7 Flex (Applied Biosystems, 4485701). This process was carried out by the Genomics Service of the IIBM facilities.

The primer pairs used for the qRT-PCR studies are listed in Additional file 1: Table S2.

The mean of three different endogenous control genes, *Prpl0,* G*apdh,* and *18 s,* was used as the internal standard for each sample. Data were expressed relative to the values obtained in WT mice (taken as 1.0) of their corresponding age after correction for the endogenous controls mean values.

### Statistical analyses

Data were expressed as mean ± SD in all figures. Graphs and statistical analyses were performed with GraphPad Prism 8 Software (www.graphpad.com). The outlier ROUT identification test (Q = 1%) was carried out for each group of data, excluding the resulting values from the final statistical analysis. For details on outlier exclusion and experimental design see Additional file 1. The estimated sample size for each animal procedure was calculated and can be found in Additional file 1: Table S3. Assessment of the normality of data was performed by Shapiro–Wilk’s test. Parametric data sets were tested using a 2-tailed, unpaired Student’s *t-*test and non-parametric data sets by Mann–Whitney’s test. Significant differences between groups were represented as * p < 0.05; **p < 0.01, *** p < 0.001 and **** p < 0.0001. All the analyses of the experimental results obtained in this work were performed in a single-blinded manner.

## Results

### *Mct8/Dio2*KO mice show NVU ultrastructural defects

**Fig. 1:**
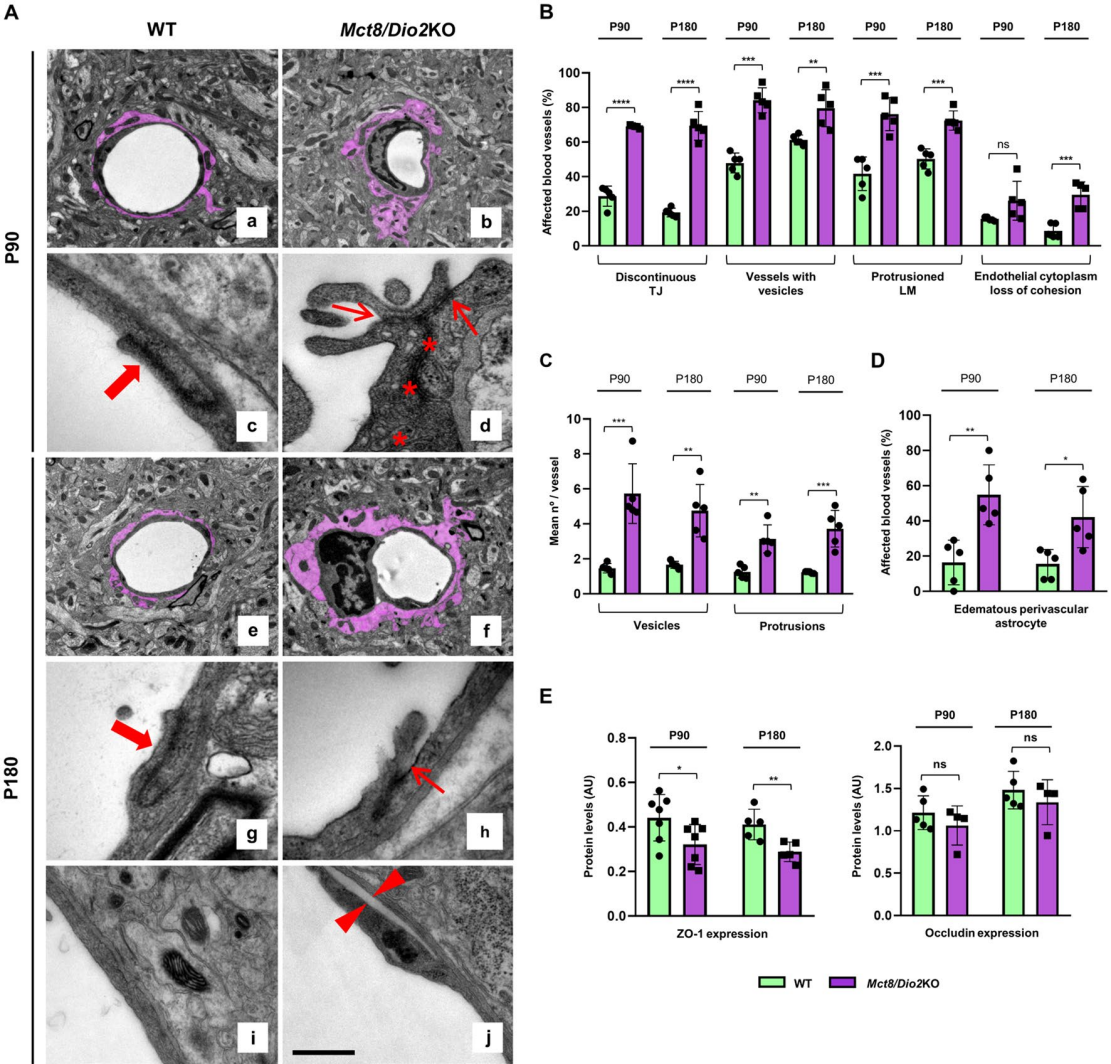
Ultrastructural analysis of the neurovascular unit (NVU) components in the cerebral cortex of WT and *Mct8/Dio2*KO mice. **A** Transmission electron microscopy (TEM) images of a representative capillary of the cerebral cortex in WT (**a** and **c** for P90; **e**, **g**, and **i** for P180) and *Mct8/Dio2*KO mice (**b** and **d** for P90; **f**, **h**, and **j** for P180). Filled arrows in **c** and **g** point at a continuous TJ. Hollow arrows in **d** and **h** point at discontinuous TJ. Asterisks in **d** mark transcytotic vesicles. Arrowheads in **j** delimit a loss of cohesion of endothelial cytoplasm. Perivascular astrocytes are colored in purple. Scale bar: 2.5 µm for **a**, **b**, **e**, and **f**; 250 nm for **c**, **d**, **g**, and **h** and 500 nm for **i** and **j**. **B** Quantification of the percentage of blood vessels showing different blood–brain barrier (BBB) disruption parameters in WT and *Mct8/Dio2*KO mice at P90 and P180. *TJ*  tight junction, *LM*  luminal membrane. **C** Quantification of the number of transcytotic vesicles and protrusions per vessel in WT and *Mct8/Dio2*KO mice at P90 and P180. **D** Quantification of the percentage of blood vessels that present an edematous perivascular astrocyte in WT and *Mct8/Dio2*KO mice at P90 and P180. **E** Western blot analysis of the expression of the main TJ proteins ZO-1 and occludin in the cerebral cortex of WT and *Mct8/Dio2*KO mice at P90 and P180. n = 5 for each experimental group for TEM studies, n = 5–7 for western blot studies at P90 and n = 5 for western blot studies at P180. Data are expressed as bar plots with individual values, mean ± SD. Assessment of the normality of data was performed by Shapiro–Wilk’s test. Parametric data sets were tested using a 2-tailed, unpaired Student’s *t*-test and non-parametric data sets by Mann–Whitney’s test. P-values *p < 0.05, **p < 0.01, ***p < 0.001, and ****p < 0.0001 were determined

To evaluate the integrity of the BBB, we used TEM to analyze the ultrastructure of the NVU in cortical vessels of the *Mct8/Dio2*KO mice compared to controls at P90 and P180 (Fig. 1A–D). The results showed that several parameters associated with BBB disruption were altered in *Mct8/Dio2*KO mice.

At both ages, the *Mct8/Dio2*KO mice presented a significantly increased percentage of discontinuous TJ in cortical vessels (Fig. 1A, arrows in panels d and h and Fig. 1B), with wide spaces in between endothelial cells compared to control mice, which showed a higher percentage of continuous TJ (Fig. 1A, filled arrows in panels c and g and Fig. 1B). Furthermore, the *Mct8/Dio2*KO mice showed an increased percentage of cortical vessels containing transcytotic vesicles (Fig. 1A, asterisks in panel d and Fig. 1B) compared to controls at both ages, as well as a significantly increased number of vesicles per vessel (Fig. 1C), which suggests an increase in the transcytotic flux in the *Mct8/Dio2*KO mice.

We also found that, at both ages, the *Mct8/Dio2*KO mice showed an increased percentage of vessels with a protruding luminal membrane (Fig. 1B), and an increased number of protrusions per vessel (Fig. 1C). No significant differences were found at either age between the width of the basal lamina of the WT mice (mean P90 width = 57.1 ± 5.4 nm, mean P180 width = 61.4 ± 11.4 nm; p-value = 0.471) and the *Mct8/Dio2*KO mice (mean P90 width = 51.2 ± 9.6 nm, mean P180 width = 55.9 ± 9.2 nm; p-value = 0.452; Additional file 1: Fig. S1A).

Moreover, at P180, it could be observed that there was a significantly increased percentage of vessels with a loss of cohesion in the endothelial cytoplasm in the *Mct8/Dio2*KO mice compared to controls, leading to a detachment of the cytoskeleton from the plasma membrane (Fig. 1A, arrowheads in panel j and Fig. 1B).

Interestingly, both at P90 and P180, there was a significant increase in the percentage of vessels with perivascular astrocytes exhibiting edema in the *Mct8/Dio2*KO mice compared to controls (Fig. 1A, highlighted in purple in panels a, b, e, and f and Fig. 1D). Nevertheless, in regard with the astrocyte end-feet (AEF), no differences in the attachment of the AEF to the endothelial cells were observed at P90 or P180 in the *Mct8/Dio2*KO mice, not even in edematous astrocytes (Additional file 1: Fig. S2).

Since different elements of the NVU were altered at an ultrastructural level, next we wanted to evaluate the expression levels of the main TJ-related proteins ZO-1 and occludin in the cerebral cortex of the *Mct8/Dio2*KO mice compared to controls. Our results show that both at P90 and P180, the *Mct8/Dio2*KO mice had a significant decrease in the expression of ZO-1 (Fig. 1E), which correlates with the ultrastructural defects observed by TEM. Occludin expression showed no differences between genotypes at both ages. Representative blots for both proteins can be found in Additional File 1: Fig. S3.

### MCT8-deficient human and mice present an increased permeability of the BBB

To further evaluate the integrity of the BBB, a series of experiments were conducted to investigate potential BBB leakage in both, MCT8-deficient human brain samples and the *Mct8/Dio2*KO mice model compared to controls.

**Fig. 2:**
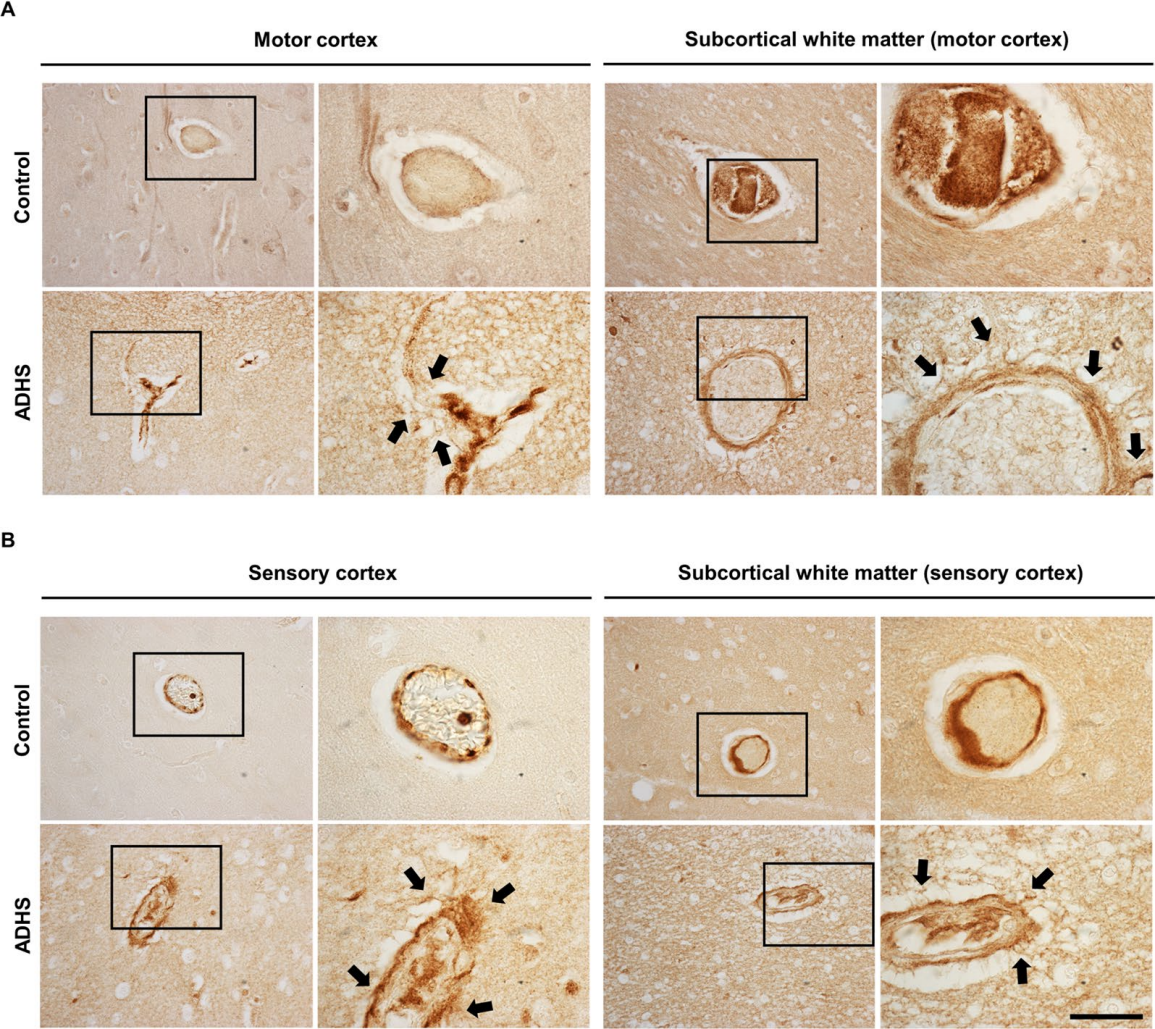
Immunodetection of IgG infiltration in the human cerebral cortex. **A** Representative microphotographs showing IgG immunodetection in the human motor cortex and motor subcortical white matter of a control subject and an MCT8-deficient subject. **B** Representative microphotographs showing IgG immunodetection in the human sensory cortex and sensory subcortical white matter of a control subject and an MCT8-deficient subject. The regions within the black boxes are shown at a higher magnification in the adjacent panel. Black arrows point at IgG-leaking blood vessels. Scale bar: 50 µm and 12.5 µm for low and high magnification images, respectively

First, for the assessment of the permeability of the BBB, the infiltration of IgG was evaluated by IHC both in human and mouse brain tissue. IgG is a protein secreted by the B lymphocytes of the periphery and it is known that, in physiological conditions, its infiltration into the brain across the BBB is highly restricted. Our results in humans showed that there was an increase in the IgG staining intensity around the blood vessels of an MCT8-deficient subject compared to a control subject in the motor cortex and in the motor subcortical white matter (Fig. 2A, arrows, 2.5 and 2.9-fold increase as compared to the control sample, respectively). This increased IgG infiltration was also observed in the sensory cortex and in the sensory subcortical white matter (Fig. 2B, arrows, 2.6 and 2.9-fold increase as compared to the control sample, respectively), suggesting extravasation of IgG at the MCT8-deficient BBB vessels.

**Fig. 3:**
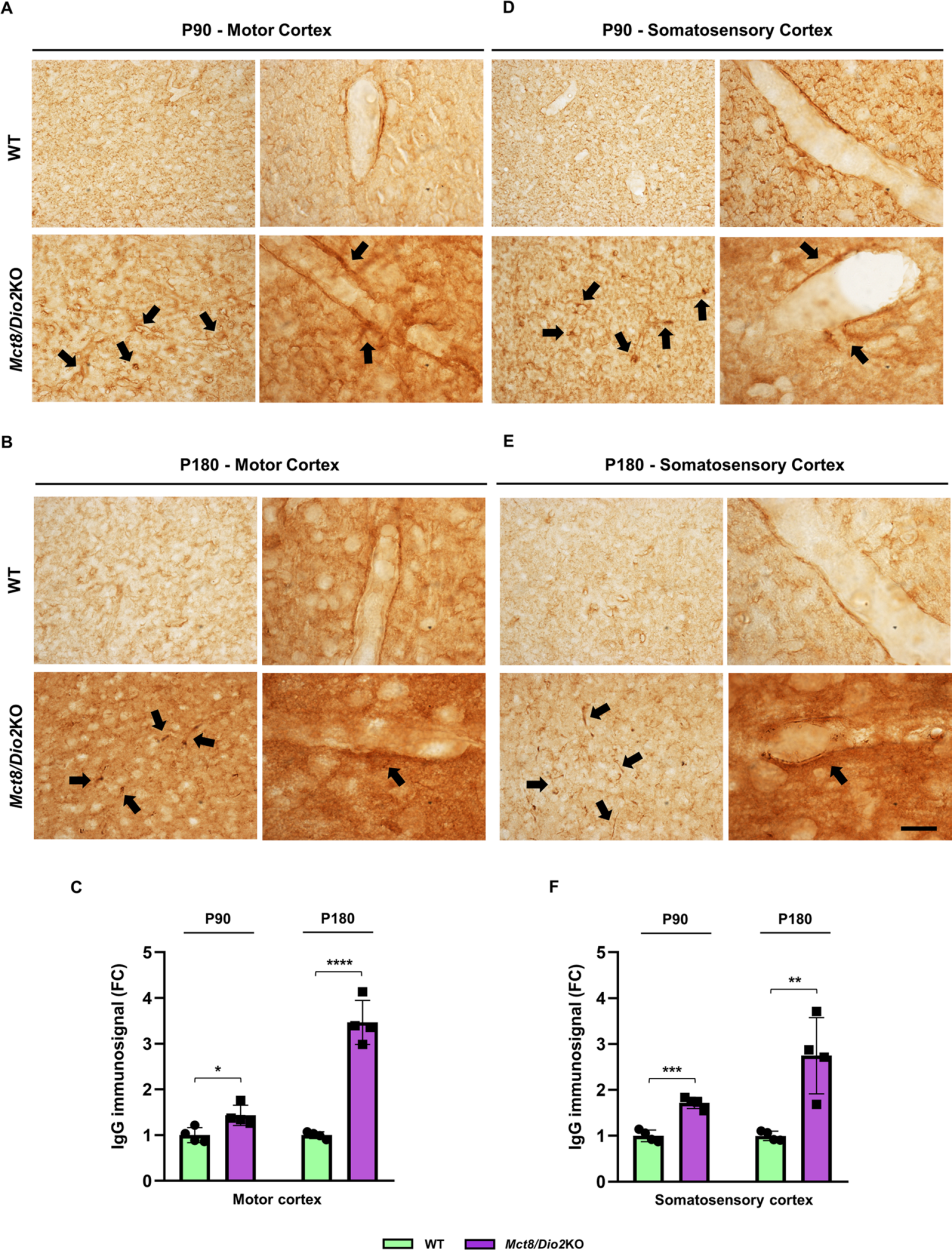
Immunodetection of IgG infiltration in the cerebral cortex of P90 and P180 WT and *Mct8/Dio2*KO mice. **A**, **B** Representative microphotographs showing IgG immunodetection in the motor cortex of WT and *Mct8/Dio2*KO mice at P90 (**A**) and P180 (**B**). **C** Quantification of IgG staining intensity in the motor cortex of WT and *Mct8/Dio2*KO mice at P90 and P180. **D**, **E** Representative microphotographs showing IgG immunodetection in the somatosensory cortex of WT and *Mct8/Dio2*KO mice at P90 (**D**) and P180 (**E**). **F** Quantification of IgG staining intensity in the somatosensory cortex of WT and *Mct8/Dio2*KO mice at P90 and P180. For all panels, left images show low magnification photographs of general parenchyma staining and right images show high magnification images of blood vessels. Black arrows point at IgG-leaking blood vessels. Scale bar: 50 µm and 20 µm for low and high magnification images, respectively. n = 4 for each experimental group of animals. *FC*  fold change. Data are expressed as bar plots with individual values, mean ± SD. P-values *p < 0.05, **p < 0.01, ***p < 0.001, and ****p < 0.0001 were determined by unpaired Student’s *t-*test

These results were replicated by the murine model of the disease. An increased IgG immunodetection was observed in the motor cortex of *Mct8/Dio2*KO mice compared to WT mice both at P90 (Fig. 3A) and P180 (Fig. 3B), being the most noticeable results found nearby the blood vessels of the *Mct8/Dio2*KO mice (arrows in Fig. 3A and B, respectively) compared to WT mice, which did not show evidence of IgG infiltration around vessels. The quantification of IgG staining validated these results, showing a statistically significant increase in the motor cortex of the *Mct8/Dio2*KO mice at both ages (Fig. 3C). The same results were observed in the somatosensory cortex of the *Mct8/Dio2*KO mice, both at P90 and P180 (Fig. 3D and E, respectively, quantified in 3F).

**Fig. 4:**
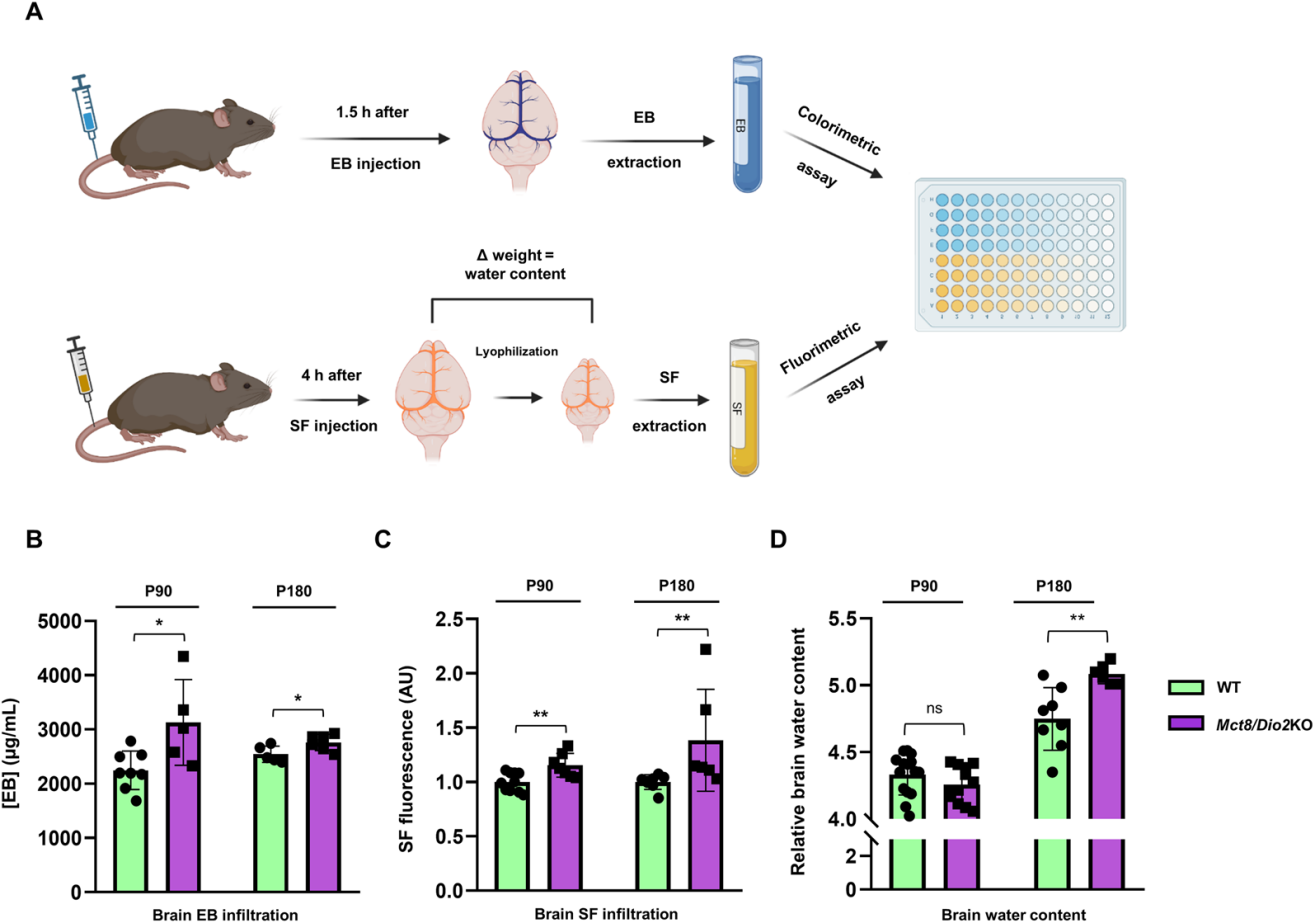
Evaluation of the infiltration of non-permeable dyes in the brain of WT and *Mct8/Dio2*KO mice. **A** Schematic representation of the protocol for Evans Blue (EB) and sodium fluorescein (SF) infiltration analysis. **B** Colorimetric quantification of the EB extracted from brains of WT and *Mct8/Dio2*KO mice at P90 and P180. For EB procedures, WT animals: n = 8 at P90 and n = 5 at P180; *Mct8/Dio2*KO animals: n = 5 at P90 and n = 8 at P180. **C** Fluorimetric quantification of the SF extracted from brains of WT and *Mct8/Dio2*KO mice at P90 and P180. For SF procedures, WT animals: n = 9 at P90 and n = 7 at P180; *Mct8/Dio2*KO animals: n = 7 at P90 and n = 6 at P180. **D** Relative brain water content of the brains of WT and *Mct8/Dio2*KO mice at P90 and P180. For brain water content calculations, WT animals: n = 14 at P90 and n = 8 at P180; *Mct8/Dio2*KO animals: n = 12 at P90 and n = 6 at P180. *EB*  Evans Blue, *SF*  sodium fluorescein, *AU*  arbitrary units. Data are expressed as bar plots with individual values, mean ± SD. P-values *p < 0.05, and **p < 0.01 were determined by unpaired Student’s *t-*test or Mann–Whitney’s test

To further corroborate this result, we also evaluated the BBB leakage in vivo by the injection and brain deposition evaluation of two different BBB non-permeable dyes: EB (Fig. 4A and B) and SF (Fig. 4A and C). The results showed that the levels of EB deposition in the brain parenchyma were significantly higher in the *Mct8/Dio2*KO mice compared to WT mice at P90 (2247 ± 353 µg/mL for WT mice and 3128 ± 789 µg/mL for *Mct8/Dio2*KO) and P180 (2545 ± 150 µg/mL for WT mice and 2761 ± 151 µg/mL for *Mct8/Dio2*KO; Fig. 4B). The levels of SF infiltration of the *Mct8/Dio2*KO mice were also higher than in the WT mice both at P90 and P180 (SF mean fluorescence was 38% higher in *Mct8/Dio2*KO mice compared to WT mice at P90 and 15% higher at P180; Fig. 4C), indicating a loss of integrity of the BBB.

Interestingly, at P180, the *Mct8/Dio2*KO mice also showed a 7% increase in the brain parenchyma water content level compared to the WT mice, indicative of brain edema (Fig. 4D), which may be related to the previous results indicating an increased leakage of the blood vessels in MCT8 deficiency.

### *Mct8/Dio2*KO mice display an increased number of brain microhemorrhages

Following the evidence of increased blood vessel permeability, we performed different histological staining techniques to evaluate the presence of microhemorrhages surrounding the vessels at the brain parenchyma as a marker of microvessel dysfunction. We have analyzed both acute or recent microhemorrhages (by means of H&E staining) and sub-acute or chronic microhemorrhages (using a Prussian blue histological staining).

**Fig. 5:**
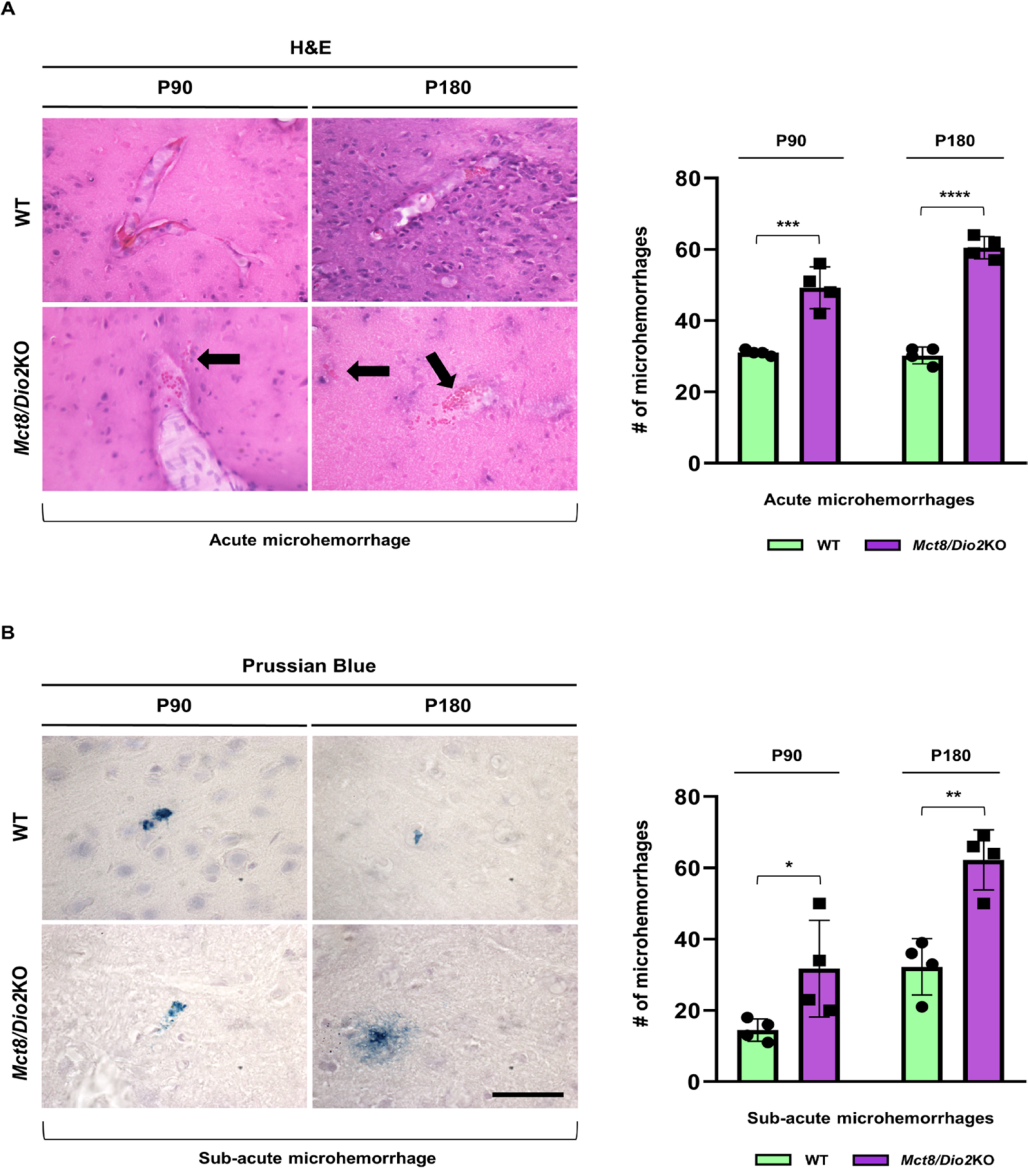
Analysis of acute and sub-acute brain microhemorrhages in WT and *Mct8/Dio2*KO mice brain. **A** Representative images of the H&E-stained brain sections from WT and *Mct8/Dio2*KO mice at P90 and P180 showing an acute microhemorrhage. Black arrows point at red blood cells leaking from a blood vessel in the *Mct8/Dio2*KO mice at both ages. The graph to the right displays a quantification of the number (#) of acute microhemorrhages stained by H&E in the whole brain parenchyma of WT and *Mct8/Dio2*KO mice at P90 and P180. **B** Representative images of the Prussian Blue-stained brain sections from WT and *Mct8/Dio2*KO mice at P90 and P180 showing a sub-acute microhemorrhage. The graph to the right displays a quantification of the number (#) of sub-acute microhemorrhages stained by Prussian Blue in the whole brain parenchyma of WT and *Mct8/Dio2*KO mice at P90 and P180. Scale bar: 50 µm for panel A and 12.5 µm for panel B. n = 4 for each experimental group. Data are expressed as bar plots with individual values, mean ± SD. P-values *p < 0.05, **p < 0.01, ***p < 0.001, and ****p < 0.0001 were determined by unpaired Student’s *t-*test

The results showed that the *Mct8/Dio2*KO mice presented an increased number of acute microhemorrhages dispersed through the whole brain parenchyma compared to controls, both at P90 and P180 (1.6-fold and twofold increase, respectively; Fig. 5A). Noticeably, erythrocyte infiltration in the brain parenchyma was observed around the blood vessels of the *Mct8/Dio2*KO mice both at P90 and P180 (Fig. 5A, arrows), but not in WT mice. Similarly, sub-acute microbleeds at brain parenchyma were also increased at both ages in *Mct8/Dio2*KO mice (2.2-fold and 1.9-fold increase, respectively; Fig. 5B) compared to controls.

### MCT8-deficient human and mice present alterations in the brain vascular network

After the structural and functional BBB alterations stated above and given that TH are directly involved in the angiogenic process and that MCT8 is expressed in the endothelial cell membrane, we wanted to evaluate if the brain vascular network was also altered both in MCT8-deficient human brain samples and the *Mct8/Dio2*KO mice.

**Fig. 6:**
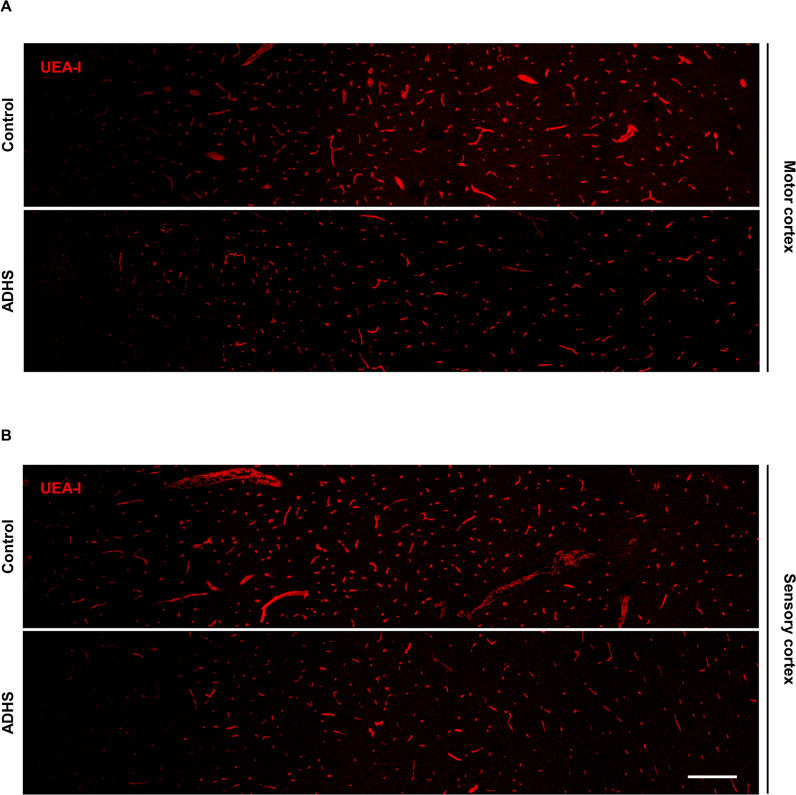
Qualitative evaluation of the blood vessel distribution in the human cerebral cortex. Representative tile confocal images of human blood vessels stained with UEA-I (*Ulex Europaeus* Agglutinin I, red) in both the motor cortex (**A**) and sensory cortex (**B**) of a control subject and an AHDS subject. Each photograph shows all cortical layers (from the meninges on the left side to the subcortical white matter on the right side). Scale bar: 250 µm

**Fig. 7:**
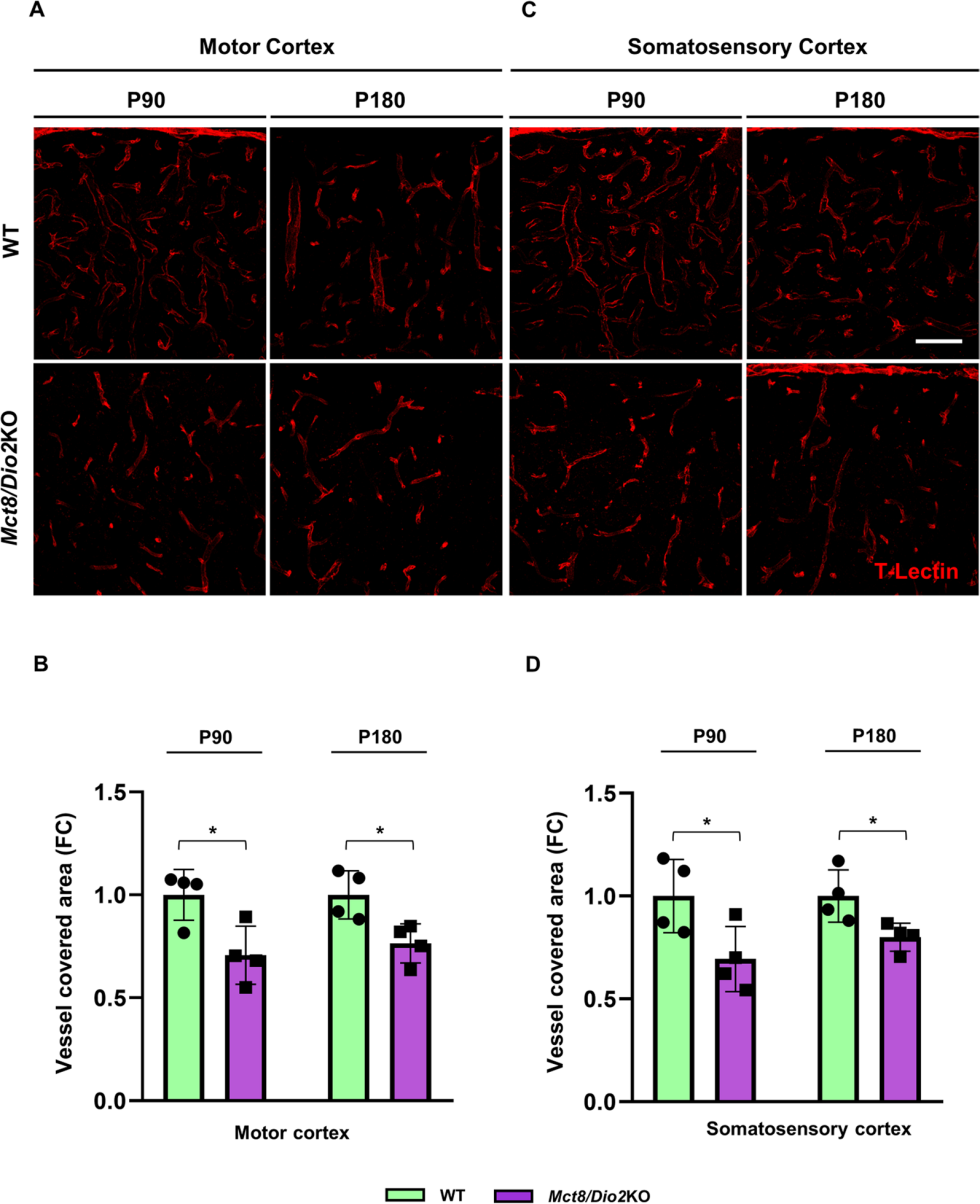
Evaluation of the blood vessel distribution in the mice cerebral cortex. **A** Representative microphotographs of mouse blood vessels stained with Tomato-Lectin (red) in the motor cortex of WT and *Mct8/Dio2*KO at P90 and P180. **B** Quantification of Tomato-Lectin staining showing vessel covered area in the motor cortex of WT and *Mct8/Dio2*KO mice at P90 and P180. **C** Representative microphotographs of mouse blood vessels stained with Tomato-Lectin (red) in the somatosensory cortex of WT and *Mct8/Dio2*KO at P90 and P180. **D** Quantification of Tomato-Lectin staining showing vessel covered area in the somatosensory cortex of WT and *Mct8/Dio2*KO mice at P90 and P180. Scale bar: 100 µm for all panels. n = 4 for each experimental group of animals. Abbreviations: FC = fold change. Data are expressed as bar plots with individual values, mean ± SD. P-values *p < 0.05 were determined by unpaired Student’s *t-*test

First, we evaluated the brain vascular density by labeling endothelial cells using fluorescent UEA-I, a gold standard method to study human endothelial cells. Our results in humans showed a reduction in the blood vessel density in both the motor cortex (Fig. 6A) and the sensory cortex (Fig. 6B) of the AHDS subject in contrast with the control subject. The AHDS tissue presented a reduction of 64.2% in UEA-I positive blood vessels at the motor cortex and a reduction of 78.0% in the sensory cortex as compared to the control subject. This result was replicated in the *Mct8/Dio2*KO mice, where Tomato-lectin was used to stain mice endothelial cells (Fig. 7). A significantly decreased blood vessel density was observed in the motor cortex (Fig. 7A, quantified in Fig. 7B) and the somatosensory cortex (Fig. 7C, quantified in Fig. 7D) compared to the WT mice both at P90 and P180.

This alteration was also noticeable in the toluidine blue stained semithin sections of the WT and *Mct8/Dio2*KO mice used for the TEM studies at P90 and P180 (Additional file 1: Fig. S1B).

To deepen into these results, we performed MRA, which is a specialized magnetic resonance imaging technique used to quantitatively assess the density of blood vessels in a specific tissue in vivo.

**Fig. 8:**
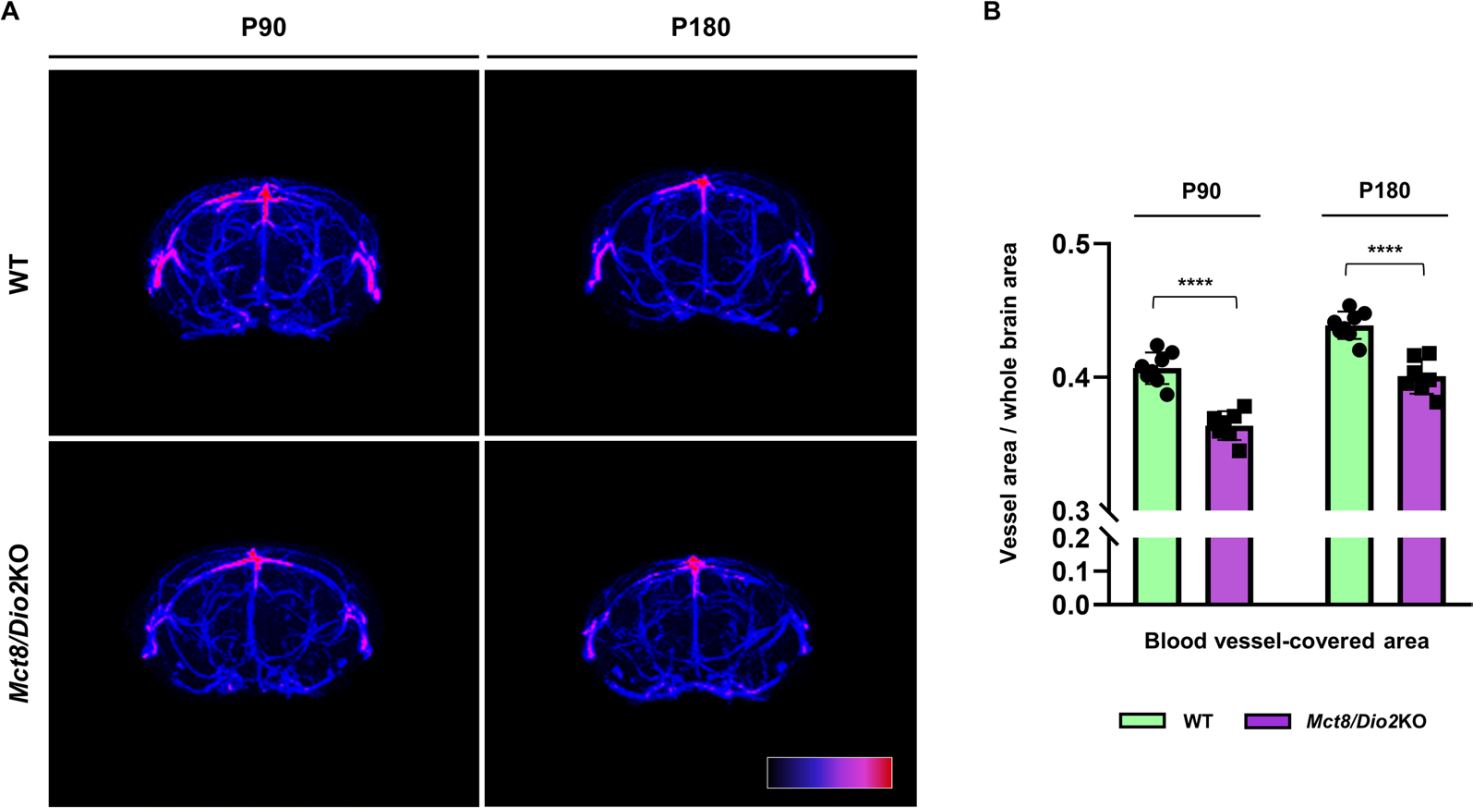
Measurement of the mean blood vessel density in the brain of WT and *Mct8/Dio2*KO mice. **A** Representative Pseudocolor Image Look-Up Table (LUT) maps from maximal intensity projections of 2D Magnetic Resonance Angiography (MRA) acquisitions of the cerebral blood-vessel network. **B** Blood-vessel covered area quantification in WT and *Mct8/Dio2*KO mice at P90 and P180. n = 10 for each experimental group. Data are expressed as bar plots with individual values, mean ± SD. P-values ****p < 0.0001 were determined by unpaired Student’s *t-*test

Thus, we run a whole-brain MRA to obtain a maximal-intensity projection of the cerebral vasculature. Then, we calculated the blood vessel-covered area in both WT and *Mct8/Dio2*KO mice at P90 and P180 (Fig. 8A). The results evidenced a significant reduction of 12% at P90 and 10% at P180 in the brain blood vessel-covered area in *Mct8/Dio2KO* mice compared to the WT mice (Fig. 8B), supporting our observations from the fluorescence staining.

### *Mct8/Dio2*KO mice show altered expression of angiogenesis-related transcription factors

**Fig. 9:**
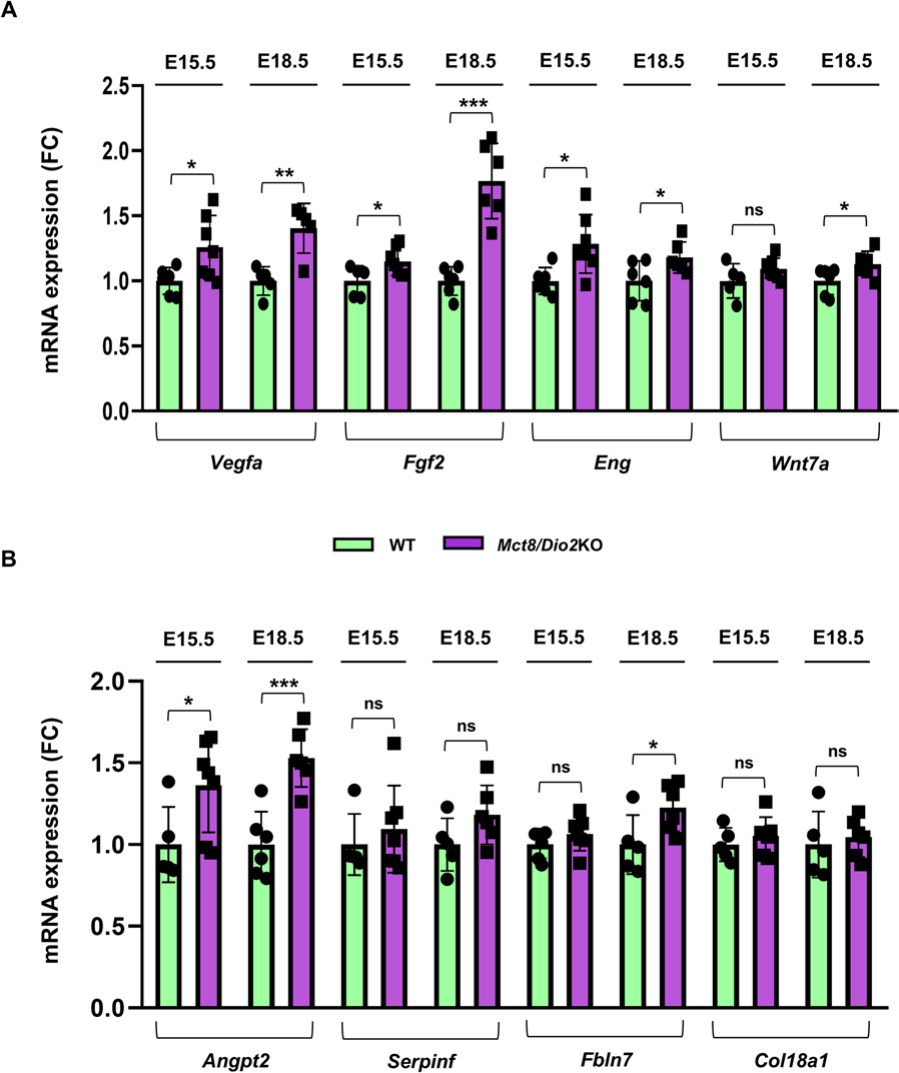
Angiogenesis-related genes expression analysis by qRT-PCR in the cerebral cortex of WT and *Mct8/Dio2*KO mice. **A** Expression of the main BBB-associated proangiogenic genes at E15.5 and E18.5: *Vegfa, Fgf2, Eng* and *Wnt7a*. **B** Expression of the main BBB-associated antiangiogenic genes at E15.5 and E18.5: *Angpt2, Serpinf, Fbln7,* and *Col18a1.* n = 6 for each experimental group at E15.5, n = 5 for E18.5 WT embryos and n = 6 for E18.5 *Mct8/Dio2*KO embryos. *Vegfa* (VEGFA, vascular endothelial growth factor type A), *Fgf2* (bFGF, fibroblast growth factor 2), *Eng* (endoglin), *Wnt7a* (WNT7A, wingless-type MMTV integration site family, member 7A), *Angpt2* (ANG-2, angiopoietin 2), *Serpinf* (PEDF, pigment epithelium-derived factor), *Fbln7* (fibulin-7), *Col18a1* (endostatin, proteolytic fragment of the collagen type XVIII protein), *FC*  fold changes. Data are expressed as bar plots with individual values, mean ± SD. P-values *p < 0.05, **p < 0.01, and ***p < 0.001 were determined by unpaired Student’s *t-*test or Mann–Whitney’s test

To gain knowledge of the potential underlying causes for the decreased blood vessel density within the brain of *Mct8/Dio2*KO mice, we performed qRT-PCR to analyze the expression levels of the main TH-regulated proangiogenic genes at critical stages of the BBB ontogeny, specifically at E15.5 and E18.5, where the formation of a mature and functional BBB is being completed. Interestingly, the results showed that both, at E15.5 and E18.5, the *Mct8/Dio2*KO mice embryos showed a significant increase in the expression of the proangiogenic genes *Vegfa*, *Fgf2,* and *Eng* (Fig. 9A)*.* Additionally, we observed an increased expression of the proangiogenic factor *Wnt7a* only at E18.5 in the *Mct8/Dio2*KO mice (Fig. 9A).

We further analyzed the angiogenic profile of these mice by evaluating the expression levels of the antiangiogenic genes *Angpt2, Serpinf, Fbln7,* and *Col18a1.* The results showed that *Angpt2* was increased in the *Mct8/Dio2*KO mice embryos at both ages examined. We also quantified an increased expression of *Fbln7* in *Mct8/Dio2*KO mice, but only at E18.5 (Fig. 9B).

## Discussion

In this work, we have focused on the study of the BBB structure and function in the context of MCT8 deficiency, to gain insight into the different pathophysiological processes involved in the neurological alterations of AHDS patients. These alterations mainly derive from the restricted TH entry to the brain due to the deficiency or altered function of the TH transporter MCT8 at the brain barriers [5, 14, 15]. Nevertheless, these alterations frequently seem to be worse than the alterations found in congenital hypothyroidism [31, 32]. The endothelial cells of the BBB (which express MCT8) are localized within an interphase between the hyperthyroid environment of the serum and the hypothyroid brain parenchyma present in MCT8 deficiency, receiving opposing signals and challenging their response. Altogether, these premises have led us to question whether the lack of MCT8 could directly induce alterations in the endothelial cells of the BBB, subsequently affecting the differentiation and function of the main components of the NVU, thereby worsening the neuropathology, and contributing to the progression of the disease.

For these studies we have analyzed brain tissue samples from an MCT8-deficient patient and brain tissue from *Mct8/Dio2*KO mice as a well-validated model for the disease [13, 26]. As stated in the introduction, these mice replicate the thyroidal profile of the patients, present several neurological and motor impairments and display myelinating alterations, being a more faithful model for AHDS than the *Mct8*KO mice. The *Mct8/Dio2*KO mice also present the advantage that only the TH availability pathway is being affected to avoid the compensatory mechanisms present in mice, in contrast to other mouse models such as the *Mct8/Oatp1c1*KO mice. The initial time point for our studies was P90, which is the corresponding age to the human sample available (an 11-year old boy, [33]), and the use of P180 mice allowed us determine if the alterations observed at P90 were persistent throughout time. The main limitation of our study is the lack of pathological human brain samples available for the different experimental procedures, being restricted to a single 11-year-old MCT8-deficient patient with limited available brain tissue sections.

We first evaluated the ultrastructural integrity of the BBB in the cerebral cortex of the *Mct8/Dio2*KO mice compared to controls. Our results revealed that several features of the endothelial cells were altered in young adult and adult *Mct8/Dio2*KO mice, such as an increased percentage of vessels with opened or discontinuous TJ, an increased number of vessels with luminal protrusions, and an increased number of protrusions per vessel, suggesting structural alterations of the NVU. In agreement with the TJ ultrastructural defects, we found significantly decreased protein levels of ZO-1, one of the main proteins that form the TJ, in the *Mct8/Dio2*KO mice both at P90 and at P180. These findings suggest a possible compromise in the *Mct8/Dio2*KO mice in one of the two main routes of trafficking across the BBB, the paracellular pathway, by which the substances cross between the endothelial cells. Regarding the second route for BBB transport, the transcytotic pathway, we observed an increased percentage of vessels with transcytotic vesicles in the luminal membrane, as well as an increased number of vesicles per vessel in the *Mct8/Dio2*KO mice. The transcytotic pathway includes receptor-mediated transcytosis (mediated by clathrin-covered vesicles) and non-specific adsorptive transcytosis [19]**.** The increase of these vesicles detected by TEM suggests an altered adsorptive transcytotic trafficking across the BBB. In physiological conditions, the paracellular pathway is highly restricted by the TJ and adherens junctions that seal the endothelial cells between each other, and the transcytotic pathway is almost limited to the receptor-mediated transcytosis to avoid the entrance to the CNS of exogenous agents. Our results suggest an alteration in both routes of trafficking, which have been associated with BBB permeability alterations, affecting the diffusion distance for solutes into the brain parenchyma [34, 35]. It has also recently been reported that moderate developmental hypothyroidism caused direct alterations in cell junction components in the neonatal ventricular endothelium, parallel to the ones described in the present work. This developmental hypothyroidism also induced downregulation of the MCT8 transporter expression in the ventricular endothelium [36]. These findings together with our results highlight the relevance of the CNS endothelial cells’ sensitivity to TH action, both by the direct TH genomic signaling and by the integrin extragenomic pathways. Previous in vitro studies using MCT8-deficient patients’ derived brain microvascular endothelial cells found no alterations in these cells as compared to control cells in transcriptome, expression of TJ proteins, or paracellular permeability [15]. However, this in vitro model comprises endothelial cells, lacking other components of the NVU. On the other hand, the reduction in ZO-1 expression at the *Mct8/Dio2*KO mice brain could be a consequence of alterations in the endothelial cells of the BBB (as other NVU components are also impaired), but we cannot exclude that this could also be due to the decreased blood vessel density that we observed in our results.

Interestingly, P180 *Mct8/Dio2*KO mice also showed a lack of cohesion of the cytoplasm of the endothelial cells, leading to a detachment of the plasma membrane from the surrounding cytoplasm. This lack of cohesion between the cytoskeleton and the plasma membrane in *Mct8/Dio2*KO mice could be due to abnormal TH levels. TH are known to regulate the biogenesis and distribution of cytoskeleton proteins during the morphological differentiation and maturation of cells [37, 38].

We have also studied by TEM the perivascular astrocytes, which terminal end-feet are a fundamental part of the NVU [19, 39]. Astrocytes are known to be direct TH target cells and are the main cells in the CNS expressing the DIO2 enzyme, located also at their end-feet [2, 40]. The results showed that the percentage of vessels with edematous perivascular astrocytes was significantly increased in the *Mct8/Dio2*KO mice at both studied ages. The signs of edema suggest an increased BBB permeability that may lead to higher water uptake by the astrocytes. It has been proven that perivascular astrocytes express MCT8 in the human motor cortex [7]. All of this suggests that the lack of MCT8 could affect other components of the NVU aside from endothelial cells, as it has been observed in other neurological diseases that course with a BBB disruption [41]. Although the presence of edematous astrocytes is increased in the cerebral cortex of *Mct8/Dio2*KO mice, the AEF appeared normal, excluding the possibility of an altered astrocyte-endothelial cell communication mechanism. Furthermore, alterations in TH levels could affect directly or indirectly the development of different types of neural cells.

All these findings strongly suggest not only structural alterations in the BBB but also compromise permeability. To confirm this hypothesis, we evaluated the infiltration levels of IgG, a peripheral immunoglobulin produced by B lymphocytes that in physiological conditions cannot cross the BBB; however, when the integrity of the BBB is compromised, increased levels of parenchymal IgG can be observed in the brain, as previously reported in other conditions associated with BBB disruption [42, 43]. We studied the infiltration of IgG in human brain tissue from an MCT8-deficient subject and a control subject. The results showed an increased intensity of IgG staining in the MCT8-deficient brain compared to the control subject in the motor cortex and especially in the sensory cortex, particularly surrounding the blood vessels and near the meninges. The murine model of the disease also showed an increased IgG infiltration both at P90 and P180 in the cerebral cortices. All these findings point to a disrupted BBB permeability in MCT8-deficient conditions both in humans and mice.

To gain insight into the permeability of the BBB, we also evaluated the infiltrating capacity of two different CNS non-permeable molecules: EB and SF. Both dyes are molecules that, after systemic administration, circulate in the bloodstream and extravasate to the peripheral tissues, although they are not able to cross the BBB in physiological conditions. Nevertheless, if the permeability of the BBB is compromised, they infiltrate into the brain parenchyma, proportionally to the degree of damage and loss of structure of the BBB; being the EB a bigger molecule than the SF (EB is transported through the bloodstream attached to albumin), thus denoting a higher degree of BBB permeability [44]. Our results proved that, at P90 and P180, both SF and EB were significantly increased in the brain parenchyma of the *Mct8/Dio2*KO mice, suggesting an increased permeability compared to control mice. More so, the SF protocol allowed us to obtain another BBB disruption-related parameter, the brain water content, which was also increased at P180 in the *Mct8/Dio2*KO mice. This has been reported to be a sign of brain edema [45], upholding the results observed in the edematous astrocytes by TEM.

To go deeper into BBB permeability, we carried out H&E and Prussian blue histological stainings, which account for acute or sub-acute microhemorrhages respectively. Both types of hemorrhages were significantly increased at P90 in the whole brain parenchyma of the *Mct8/Dio2*KO mice compared to controls. This result was also observed, and slightly increased, at P180, as it has been reported that cerebral microbleeds physiologically increase with aging [46]. These findings confirm an increased vessel leakage in the *Mct8/Dio2*KO mice which progressively worsens with age. Similar observations of vascular leakage have been reported in other CNS hematological disorders, including stroke and inflammation-induced BBB disruption [47, 48].

A crucial point of discussion is whether the BBB disruption in the absence of MCT8 is able to mediate transport of TH into the brain. This would partly explain why *Mct8/Dio2*KO mice, despite lacking T3 transport through MCT8 and conversion of T4 into T3 by DIO2, exhibit a cerebral cortex T3 content that is only half of that observed in WT values [26]. Similar findings were observed in the T3 brain content of an MCT8-deficient fetus [8]. As to why TH leakage into the brain through the disrupted BBB is not able to fully compensate for the lack of MCT8, we propose the following speculations. First, TH circulate in the bloodstream bound to carrier proteins, mainly thyroxine-binding globulin, transthyretin, and albumin [49], with only a small fraction of TH circulating in their free form. All of these carrier proteins are large molecules with complex tridimensional structures that would not allow for the TH-transporter protein complex to cross through the disrupted endothelial cells. Another possibility could be that the mechanisms that maintain the homeostasis of TH in the endothelial cells, mainly DIO3 enzymatic activity, could be preventing TH from trespassing through the disrupted BBB. DIO3 enzyme is responsible for the inactivation of T4 and T3 by deiodination into their inactive forms rT3 and T2, respectively. Studies by Vatine et al. [15] revealed that *DIO3,* not only is highly expressed at the endothelial cells of the BBB, but its expression further increases in MCT8-deficient patient derived brain endothelial cells, suggesting that this protein plays an important role in the TH metabolism at the BBB-endothelial cell level. Moreover, it has been suggested that the catalytic site of this protein is on the extracellular domain [50], thereby potentially facilitating the inactivation of TH that could be crossing through the disrupted BBB.

Expanding the focus to a general view of the brain blood vessel network, we evaluated the brain vasculature both in human and mice brain samples. The AHDS subject showed a decreased vessel density in the motor cortex and sensory cortex compared to the control subject; which was replicated by the *Mct8/Dio2*KO mice both at P90 and P180. We also quantitatively measured the blood vessel density in the mouse brain by MRA. *Mct8/Dio2*KO mice showed a significant decrease in the vessel-covered area of the brain compared to controls at both ages of study, suggesting an angiogenic defect in the *Mct8/Dio2*KO brain, as reported in other neurological alterations studied by MRA such as thromboembolic stroke or aneurisms [51–53]. For these studies we used TOF-MRA, a translational radiodiagnostic method commonly employed in clinical settings to assess vascular integrity in a fast and easy procedure [54]. The resolution of this procedure allows a robust estimation of the intracranial main brain vasculature (vessels with diameters around 100 μm or larger) and has demonstrated enough sensitivity to detect alterations in *Mct8/Dio2*KO mice blood vessel density.

To confirm or discard angiogenic alterations as a pathophysiological mechanism in MCT8 deficiency, we measured the level of expression of different proangiogenic and antiangiogenic genes at the critical periods for the ontogeny of the BBB: E15.5, when TJ stop showing transcytotic vesicles and peripheral tracer infiltration is no longer visible in the mouse brain [55], and E18.5, when identifiable closed TJ are first observed [19, 56]. The main known BBB-associated proangiogenic factors, *Vegfa, Fgf2, Eng,* and *Wnt7a,* were significantly increased in *Mct8/Dio2*KO embryos compared to controls. We also observed increased levels of expression of the antiangiogenic genes *Angpt2* and *Fbln7*. The signaling of TH is known to be an important regulator of the expression of angiogenic factors, mainly through the extragenomic actions of T4 [23, 57]. The increased levels in both pro- and anti-angiogenic factors found in *Mct8/Dio2*KO embryos denote an imbalance of the angiogenic process fine regulation, causing alterations in the vascular remodeling that might lead to the decreased blood-vessel density observed at adult stages in these mice. Further studies are needed to gain insight into the relevance of the combined alterations in the pro- and anti-angiogenic factors found in this animal model, as well as their impact on pruning and/or vessel remodeling to contribute to our understanding of these alterations.

It remains unknown, however, why the disruption of the integrity of the BBB that we describe in these mice is not allowing the entrance of peripheral TH into the brain parenchyma, as the *Mct8/Dio2*KO mice brain are known to be hypothyroid at adult stages [26]. It is important to consider that MCT8 deficiency is a disease with a unique hormonal profile for the endothelial cells of the BBB, given the fact that the luminal side is in contact with a hyperthyroid serum, but the abluminal membrane is facing the hypothyroid brain parenchyma, being possibly exposed to contradictory inputs from both sides that may cause all the alterations in the different pathways described in this paper. The specific pathophysiological mechanisms by which these alterations in the BBB integrity are caused, however, remain to be discovered. This double-sided exposure to TH content was also described by O’Shaughnessy et al. for the endothelial cells of the subventricular zone, where the luminal cells are in contact with the cerebrospinal fluid, whereas the ventricular epithelium is enriched in BBB vascular networks, residing these cells in an intersection for TH transport, and exhibiting a striking spatiotemporal TH sensitivity during the perinatal period [36].

## Conclusions

To sum up, this study describes for the first time that MCT8 deficiency leads to BBB structural abnormalities in vivo subsequently leading to impaired endothelial cell function, as demonstrated in the mouse model of the disease and the available MCT8-deficient human brain samples. This newly described pathophysiological mechanism of the disease opens a new field of study in terms of the relevance of TH transporters in the development and maintenance of the BBB. Furthermore, it underscores the influence of MCT8 on the functionality of the NVU and endothelial cells per se. This might be a new key point in the study of AHDS and other neurological disorders related to membrane transporters, allowing this new pathophysiological mechanism to be explored for possible therapies.

### Supplementary Information

**Additional file 1: Figure S1.** Cortical blood vessel basal lamina width measurement and semithin sections of WT and *Mct8/Dio2*KO mice brain. A. Quantification of the basal lamina width of the cortical blood vessels at P90 and P180. B. Representative Toluidine Blue-stained semithin sections from WT and *Mct8/Dio2*KO mice at P90 and P180. Scale bar: 200 μm. n = 5 for each experimental group. Data are expressed as bar plots with individual values, mean ± SD. P-values were determined by unpaired Student’s t-test or Mann-Whitney’s test showing no statistical differences. **Figure S2.** Ultrastructural analysis of the astrocyte end feet (AEF) coverage of the blood vessels in P90 and P180 WT and *Mct8/Dio2*KO mice. A. Transmission electron microscopy (TEM) images of representative capillaries of the cerebral cortex in WT and *Mct8/Dio2*KO mice at P90 and P180. Perivascular astrocytes are colored in purple. Scale bar: 2.5 μm. Abbreviations: AEF = astrocyte end feet. B. Quantification of the percentage of blood vessels that present a detached AEF in WT and *Mct8/Dio2*KO mice at P90 and P180. n = 5 for each experimental group. Data are expressed as bar plots with individual values, mean ± SD. P-values were determined by unpaired Student’s t-test showing no statistical differences. **Figure S3.** Western blot representative blots for ZO-1 and occludin protein expression in the cortex of P90 and P180 WT and *Mct8/Dio2*KO mice. A. Western blot analysis of the expression of the tight junction protein ZO-1 in the cerebral cortex of WT and *Mct8/Dio2*KO mice at P90 and P180. B. Western blot analysis of the expression of the tight junction protein occludin in the cerebral cortex of WT and *Mct8/Dio2*KO mice at P90 and P180. **Table S1.** BRISQ guidelines checklist for human samples relevant to the present study. **Table S2.** Sequences of forward and reverse primers used in qRT-PCR. **Table S3.** EDA-NC3Rs sample size calculations.

## Data Availability

All data generated or analyzed during this study are included in this published article and its additional information files.

## Abbreviations

AEF: Astrocyte end-feet
AHDS: Allan-Herndon-Dudley Syndrome
*Angpt2*: Angiopoietin 2
BBB: Blood–brain barrier
CNS: Central nervous system
DIO2: Deiodinase type 2
E15.5/E18.5: Embryonic day 15.5/18.5
EB: Evans blue
F*gf2*: Fibroblast growth factor 2
IHC: Immunohistochemistry
MCT8: Monocarboxylate transporter 8
MRA: Magnetic resonance angiography
NA: Numerical aperture
NVU: Neurovascular unit
P90/P180: Postnatal day 90/180
PB: Phosphate buffer
ROI: Region of interest
SF: Sodium fluorescein
T3: Triiodothyronine
T4: Thyroxine
TEM: Transmission electron microscopy
TJ: Tight junction
TH: Thyroid hormone
V*egfa*: Vascular endothelial growth factor A
WB: Western blot
W*nt7a*: Wingless-type MMTV integration site family, member 7A
ZO-1: Zonula occludens 1
