# Supplementary material for: Neurovascular unit disruption and blood–brain barrier leakage in MCT8 deficiency
Source: Fluids Barriers CNS. 2023 Nov 3;20:79. doi: 10.1186/s12987-023-00481-w (PMC10623792; doi:10.1186/s12987-023-00481-w)
Supplement: Supplementary file 1 — Additional file 1: Figure S1. Cortical blood vessel basal lamina width measurement and semithin sections of WT and Mct8/Dio2KO mice brain. A. Quantification of the basal lamina width of the cortical blood vessels at P90 and P180. B. Representative Toluidine Blue-stained semithin sections from WT and Mct8/Dio2KO mice at P90 and P180. Scale bar: 200 μm. n = 5 for each experimental group. Data are expressed as bar plots with individual values, mean ± SD. P-values were determined by unpaired Student’s t-test or Mann-Whitney’s test showing no statistical differences. Figure S2. Ultrastructural analysis of the astrocyte end feet (AEF) coverage of the blood vessels in P90 and P180 WT and Mct8/Dio2KO mice. A. Transmission electron microscopy (TEM) images of representative capillaries of the cerebral cortex in WT and Mct8/Dio2KO mice at P90 and P180. Perivascular astrocytes are colored in purple. Scale bar: 2.5 μm. Abbreviations: AEF = astrocyte end feet. B. Quantification of the percentage of blood vessels that present a detached AEF in WT and Mct8/Dio2KO mice at P90 and P180. n = 5 for each experimental group. Data are expressed as bar plots with individual values, mean ± SD. P-values were determined by unpaired Student’s t-test showing no statistical differences. Figure S3. Western blot representative blots for ZO-1 and occludin protein expression in the cortex of P90 and P180 WT and Mct8/Dio2KO mice. A. Western blot analysis of the expression of the tight junction protein ZO-1 in the cerebral cortex of WT and Mct8/Dio2KO mice at P90 and P180. B. Western blot analysis of the expression of the tight junction protein occludin in the cerebral cortex of WT and Mct8/Dio2KO mice at P90 and P180. Table S1. BRISQ guidelines checklist for human samples relevant to the present study. Table S2. Sequences of forward and reverse primers used in qRT-PCR. Table S3. EDA-NC3Rs sample size calculations. [file 12987_2023_481_MOESM1_ESM.pdf]

Neurovascular unit disruption and blood-brain barrier leakage in MCT8 deficiency.

Marina Guillén-Yunta[1], Víctor Valcárcel-Hernández[1], Ángel García-Aldea[1], Guadalupe Soria[2], José Manuel García-Verdugo[3], Ana Montero-Pedrazuela[1], Ana Guadaño-Ferraz[1]

[1]Laboratory of Thyroid hormones and CNS. Department of Endocrine and Nervous System Pathophysiology. Instituto de Investigaciones Biomédicas 'Alberto-Sols', Consejo Superior de Investigaciones Científicas (CSIC)-Universidad Autónoma de Madrid (UAM), 28029 Madrid, Spain.

[2]Laboratory of Surgical and Experimental Neuroanatomy, Faculty of Medicine and Health Sciences, Institute of Neurosciences, University of Barcelona, Spain.

[3]Laboratory of Comparative Neurobiology, Cavanilles Institute of Biodiversity and Evolutionary Biology and Department of Cellular Biology, University of Valencia and CIBERNED-ISCIII, Valencia, Spain.

Corresponding authors:

Ana Montero-Pedrazuela, amontero@iib.uam.es. Phone: +34 91 585 4465.

Ana Guadaño-Ferraz, ana.guadano.ferraz@csic.es. Phone: +34 91 585 4494.

Journal name: Fluids and Barriers of the CNS

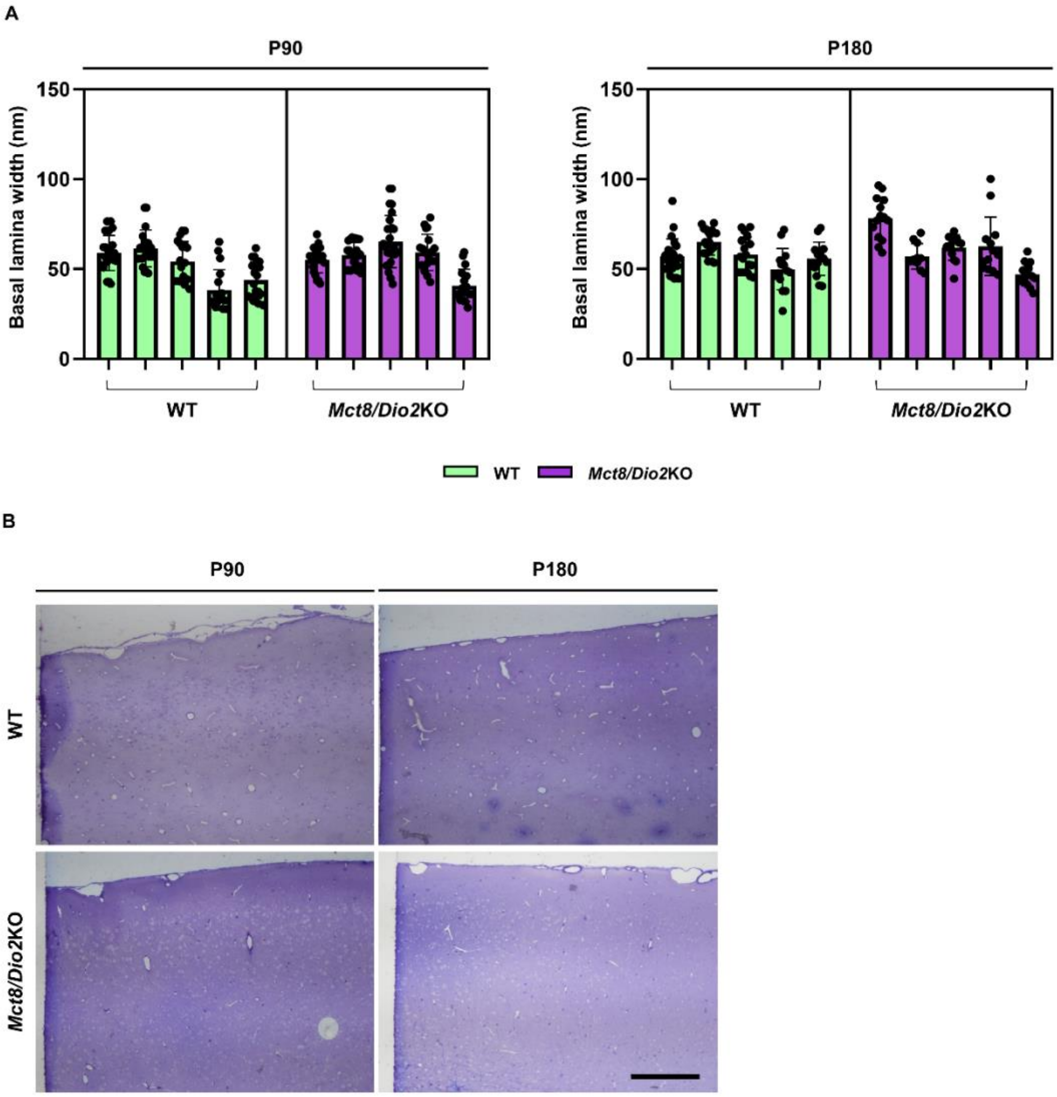

Figure S1. Cortical blood vessel basal lamina width measurement and semithin sections of WT and *Mct8/Dio2*KO mice brain. A. Quantification of the basal lamina width of the cortical blood vessels at P90 and P180. **B.** Representative Toluidine Blue-stained semithin sections from WT and *Mct8/Dio2*KO mice at P90 and P180. Scale bar: 200 µm. n = 5 for each experimental group. Data are expressed as bar plots with individual values, mean ± SD. P-values were determined by unpaired Student's *t*-test or Mann-Whitney's test showing no statistical differences.

A

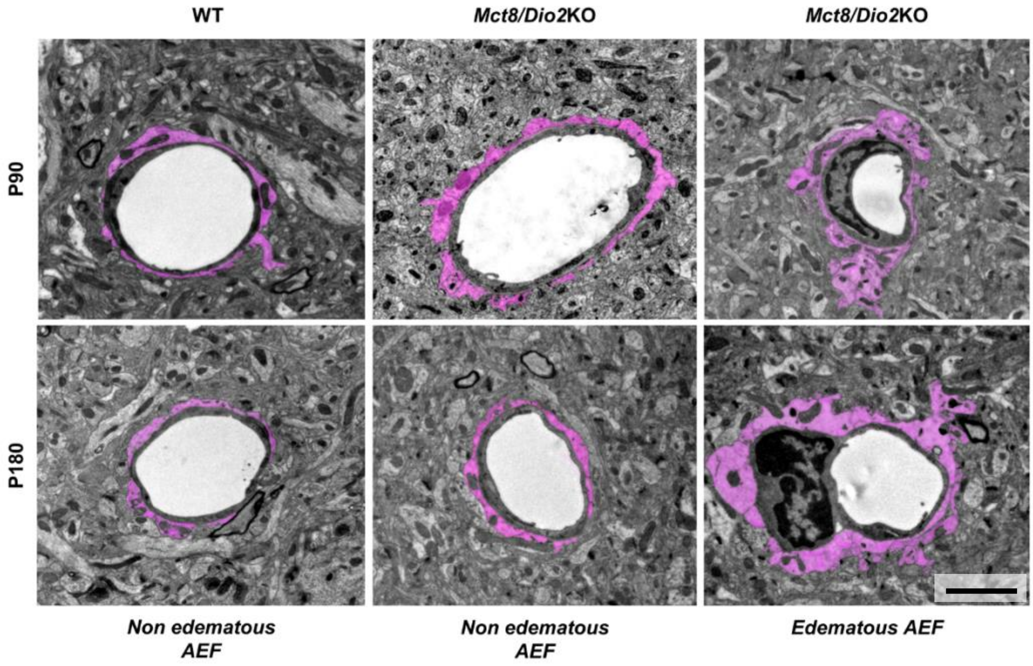

B

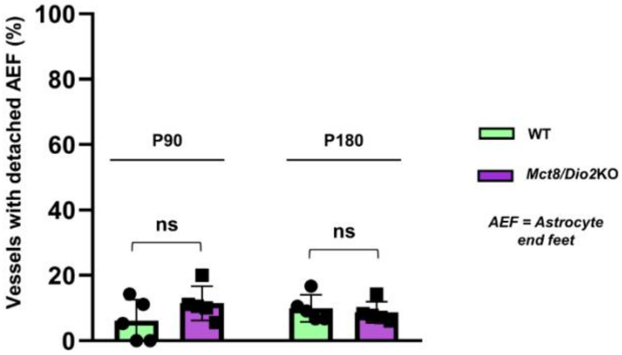

Figure S2. Ultrastructural analysis of the astrocyte end feet (AEF) coverage of the blood vessels in P90 and P180 WT and *Mct8/Dio2*KO mice. A. Transmission electron microscopy (TEM) images of representative capillaries of the cerebral cortex in WT and *Mct8/Dio2*KO mice at P90 and P180. Perivascular astrocytes are colored in purple. Scale bar: 2.5 µm. Abbreviations: AEF = astrocyte end feet. **B.** Quantification of the percentage of blood vessels that present a detached AEF in WT and *Mct8/Dio2*KO mice at P90 and P180. n = 5 for each experimental group. Data are expressed as bar plots with individual values, mean ± SD. P-values were determined by unpaired Student's *t*-test showing no statistical differences.

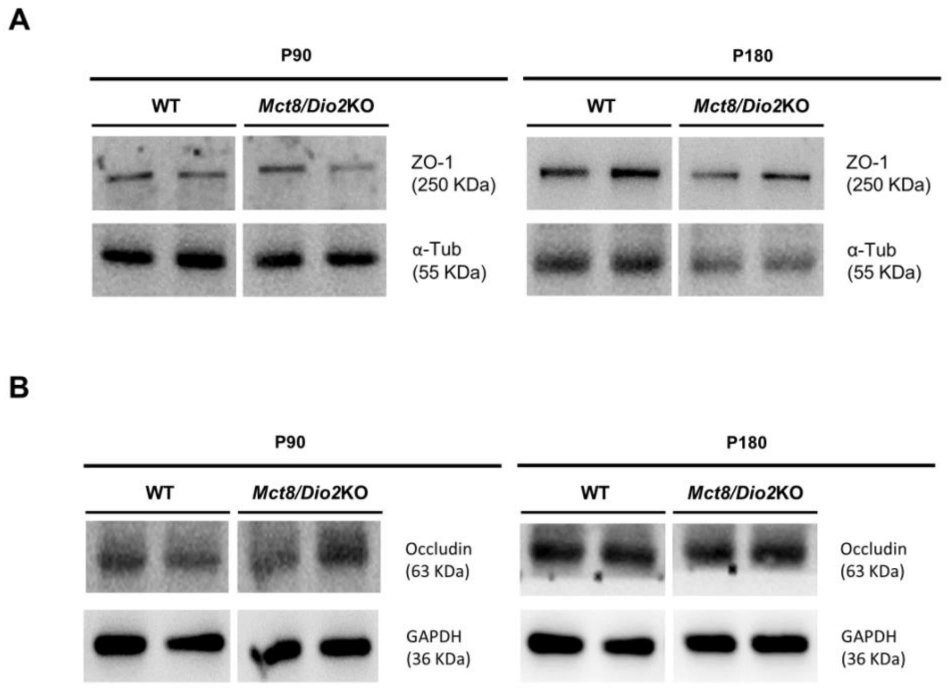

Figure S3. Western blot representative blots for ZO-1 and occludin protein expression in the cortex of P90 and P180 WT and *Mct8/Dio2*KO mice. A. Western blot analysis of the expression of the tight junction protein ZO-1 in the cerebral cortex of WT and *Mct8/Dio2*KO mice at P90 and P180. **B.** Western blot analysis of the expression of the tight junction protein occludin in the cerebral cortex of WT and *Mct8/Dio2*KO mice at P90 and P180.

Table S1. ***BRISQ guidelines checklist for human samples relevant to the present study***

	Subject of study	
Parameter	**MCT8-deficient subject**	**Control subject**
Biospecimen type	Brain tissue	Brain tissue
Anatomical site	Motor cortex, sensory cortex	Motor cortex, sensory cortex
Clinical diagnosis	Allan-Herndon-Dudley Syndrome (AHDS) (*SLC16A2* mutation Q96X)	Healthy control
Clinical characteristics	Severe psychomotor developmental delay, seizures, reduced myelination and AHDS characteristic thyroid profile	No central nervous system-associated pathology, normal thyroid profile
Vital state	Deceased	Deceased
Cause of death	Respiratory failure secondary to aspiration pneumonia	Diffuse lymphocytic myocarditis
Collection mechanism	Postmortem necropsy	Postmortem necropsy
Long term preservation	Tissue blocks fixed in formalin and embedded in paraffin	Tissue blocks fixed in formalin and embedded in paraffin
Sample source	Sydney Children's and Prince of Wales Hospital in Randwick, Australia	L'Hospital Infantil Sant Joan de Déu Biobank, Barcelona, Spain

Table S1. BRISQ guidelines checklist for human samples used for the present study. This table lists several parameters for the human biospecimens employed in this study, along with the specific traits for these parameters for the control and MCT8-Deficient subjects.

Table S2. ***Sequences of forward and reverse primers used in qRT-PCR***

Gene (Protein)	Primer, 5’-3’ Forward	Reverse
Vegfa (VEGFA)	CTTGTTCAGAGCGGAGAAAGC	ACATCTGCAAGTACGTTCGTT
Fgf2 (bFGF)	GTCAAACTACAACTCCAAGCAG	GAAACACTCTCCTGTAACACACTT
Eng (endoglin)	CGATAGCAGCACTGGATGAC	AGAATGGTGCCTTTGGGTCT
Wnt7a (WNT7A)	CCGAAATGGCCGTTGG	CGATGCCGTAGCGGATGT
Angpt2 (ANG-2)	CCAACTCCAAGAGCTTCGGTT	CGGTGTTGGATGACTGTCCA
Serpinf1 (PEDF)	AGGCGAACTTACCAAGTCTCTG	TGTTCCACTTGGGTGAGCTT
Fbln7 (fibulin-7)	GTGGATACCGGATCCTGGCTGAT	ACTCAGGGTTGACACACTGGAA
*Col18a1** (endostatin)	ACAGTTGCTGCCTCAGACCT	CAGTCAGGAGAGCTGGTTCC

** Endostatin is a proteolytic fragment of the collagen type XVIII protein*

Table S2. Sequences of forward and reverse primers used in qRT-PCR. The table provides a comprehensive list of the genes studied in the qRT-PCR analyses, along with their corresponding encoded proteins enclosed in brackets. Additionally, the sequences of the primers used for each gene in the 5’-3’ sense and the NCBI accession number of their mRNA used as a template for the primer design, are also included.

DETAILS FOR THE EXPERIMENTAL DESIGN

The experimental design for each procedure involving animal experimentation was carried out following the ARRIVE guidelines (Percie du Sert et al. 2020), as follows:

1. Study design:

a. The groups compared were:

In the electron microscopy, western blot, immunohistochemistry, non-permeable dye injections, histological staining procedures, immunofluorescence and MRA: (1) WT and *Mct8/Dio2*KO mice at P90 and (2) WT and *Mct8/Dio2*KO mice at P180.

In gene expression analyses: (1) WT and *Mct8/Dio2*KO fetuses at E15.5 and (2) WT and *Mct8/Dio2*KO fetuses at E18.5.

b. The experimental unit was single mice for all the experimental procedures listed in section 1a.

2. Sample size, inclusion and exclusion criteria:

a. Estimated sample size for each experiment was calculated using the power calculation tool from the Experimental Design Assistant tool (EDA) of the National Centre for the Replacement Refinement and Reduction of Animals in Research (NC3Rs) (PMID: 28957312). For all experiments, a significance threshold (α) of 0.05 and a statistical power ($1-\beta$) of 0.95 were used, introducing the specific variability of the WT group and the effect size for each experiment. The summary of the estimated sample size for each animal procedure is shown in Supplementary Table 3 (see below).

b. Only males were used for the experiments as MCT8 Deficiency is an X-linked disease.

c. All animals were included for every analysis apart from those identified as outliers by ROUT analysis (Q=1%), except for the non-permeable dye injections, where some animals were excluded from the study due to technical tail vein injection problems.

The exact value of n in each experimental group after outlier exclusion is indicated as well as the total outliers excluded in brackets:

Electron microscopy studies: n=5 for P90 WT and *Mct8/Dio2*KO and n=5 for P180 WT and *Mct8/Dio2*KO mice. No outliers.

Immunohistochemistry: n=4 for P90 and P180 WT and *Mct8/Dio2*KO mice. No ourliers.

Histological staining procedures: n=4 for P90 and P180 WT and *Mct8/Dio2*KO mice. No outliers.

Evans Blue injections: n=8 P90 WT, n=5 (2 outliers, 1 excluded animal) for P90 *Mct8/Dio2*KO, n=5 (2 outliers, 1 excluded animal) P180 WT and n=8 for P180 *Mct8/Dio2*KO mice.

Sodium Fluorescein injections: n=9 (1 outlier) P90 WT, n=7 (2 outliers, 1 excluded animal) for P90 *Mct8/Dio2*KO, n=7 (1 outlier) P180 WT and n=6 (2 outliers, 1 excluded animal) for P180 *Mct8/Dio2*KO mice.

Water content calculation: n=14 P90 WT, n=12 for P90 *Mct8/Dio2*KO, n=8 (2 outliers) P180 WT and n=6 (3 outliers) for P180 *Mct8/Dio2*KO mice.

Blood vessel labeling: n=4 for P90 and P180 WT and *Mct8/Dio2*KO mice. No outliers.

MRA studies: n=8 for P90 and P180 WT and *Mct8/Dio2*KO mice. No outliers.

Semithin sections studies: n=6 for P90 WT and *Mct8/Dio2*KO and n=5 (1 outlier) for P180 WT and n=6 for *Mct8/Dio2*KO mice.

Gene expression studies: n=6- (1 outlier) for E15.5 WT and *Mct8/Dio2*KO embryos, n=6 for E18.5 WT and *Mct8/Dio2*KO embryos.

3. Randomization

a. Animal cage location was decided using https://www.random.org/lists/

b. Animals were genotyped to distinguish *Mct8*$^{+/-}$*Dio2*$^{-/-}$ mice from WT mice. Once the genotype was identified, non-permeable dye injection, MRA study and tissue collection order were decided in a randomized manner between both groups by flipping a coin.

4. Blinding

Researchers were single blinded during the data analysis for all the experimental procedures listed above.

Table S3. ***EDA-NC3Rs estimated sample size for each animal procedure***

Experimental procedure	Effect size	STD	Significance level	Power	Estimated n per group
Electron microscopy	20.8	4.125	0.05	0.9	3
Western Blot	-0.123	0.067	0.05	0.9	8
Evans Blue injections	880.9	353	0.05	0.9	5
Sodium Fluorescein injections	0.154	0.086	0.05	0.9	8
H&E staining	18.25	0.817	0.05	0.9	2
Prussian Blue staining	17.25	3.109	0.05	0.9	3
Magnetic Resonance Angiography	-0.043	0.012	0.05	0.9	3
Gene expression	0.312	0.107	0.05	0.9	4

Table S3. ***EDA-NC3Rs*** **sample size calculations.** In this table are listed the size effect, standard deviation, significance threshold, statistical power, and estimated sample size (n) per group calculated for each quantitative animal procedure carried out in this study calculated using the power calculation tool from the Experimental Design Assistant tool (EDA) of the National Centre for the Replacement Refinement and Reduction of Animals in Research (NC3Rs).
